# Activation of transcription factor CREB in human macrophages by *Mycobacterium tuberculosis* promotes bacterial survival, reduces NF-kB nuclear transit and limits phagolysosome fusion by reduced necroptotic signaling

**DOI:** 10.1371/journal.ppat.1011297

**Published:** 2023-03-31

**Authors:** Chrissy M. Leopold Wager, Jordan R. Bonifacio, Jan Simper, Adrian A. Naoun, Eusondia Arnett, Larry S. Schlesinger

**Affiliations:** 1 Host Pathogen Interaction Program, Texas Biomedical Research Institute, San Antonio, Texas, United States of America; 2 Medical Scientist Training Program, Department of Microbiology, Immunology and Molecular Genetics, UT Health Science Center San Antonio, San Antonio, Texas, United States of America; 3 Department of Biology, The University of Texas at San Antonio, San Antonio, Texas, United States of America; University of Washington, UNITED STATES

## Abstract

Macrophages are a first line of defense against pathogens. However, certain invading microbes modify macrophage responses to promote their own survival and growth. *Mycobacterium tuberculosis* (*M*.*tb*) is a human-adapted intracellular pathogen that exploits macrophages as an intracellular niche. It was previously reported that *M*.*tb* rapidly activates cAMP Response Element Binding Protein (CREB), a transcription factor that regulates diverse cellular responses in macrophages. However, the mechanism(s) underlying CREB activation and its downstream roles in human macrophage responses to *M*.*tb* are largely unknown. Herein we determined that *M*.*tb*-induced CREB activation is dependent on signaling through MAPK p38 in human monocyte-derived macrophages (MDMs). Using a CREB-specific inhibitor, we determined that *M*.*tb*-induced CREB activation leads to expression of immediate early genes including COX2, MCL-1, CCL8 and c-FOS, as well as inhibition of NF-kB p65 nuclear localization. These early CREB-mediated signaling events predicted that CREB inhibition would lead to enhanced macrophage control of *M*.*tb* growth, which we observed over days in culture. CREB inhibition also led to phosphorylation of RIPK3 and MLKL, hallmarks of necroptosis. However, this was unaccompanied by cell death at the time points tested. Instead, bacterial control corresponded with increased colocalization of *M*.*tb* with the late endosome/lysosome marker LAMP-1. Increased phagolysosomal fusion detected during CREB inhibition was dependent on RIPK3-induced pMLKL, indicating that *M*.*tb*-induced CREB signaling limits phagolysosomal fusion through inhibition of the necroptotic signaling pathway. Altogether, our data show that *M*.*tb* induces CREB activation in human macrophages early post-infection to create an environment conducive to bacterial growth. Targeting certain aspects of the CREB-induced signaling pathway may represent an innovative approach for development of host-directed therapeutics to combat TB.

## Author summary

*Mycobacterium tuberculosis* (*M*.*tb*) targets macrophages that are not well equipped to kill this host-adapted bacterium. Infection can result in development of tuberculosis (TB), a leading cause of death worldwide. Access to treatment and prevention has been compromised due to the COVID-19 pandemic, leading to the first uptick in reported cases in over a decade. *M*.*tb* has adapted to living within human macrophages, co-opting cell-signaling pathways to evade the immune response. Here, we identify cAMP Response Element Binding Protein (CREB) as a transcription factor activated by *M*.*tb* to regulate several key factors that culminate in promoting bacterial growth in human macrophages. We distinguish upstream signaling events required for CREB activation by *M*.*tb* as well as downstream effector functions including reduced NF-kB nuclear localization and phagosome-lysosome fusion. Furthermore, we show that CREB regulates bacterial trafficking by inhibiting phosphorylation of RIPK3 and MLKL, proteins important in the necroptotic cell death pathway, without influencing cell death. Altogether, we demonstrate that CREB activation is key for immune evasion by *M*.*tb* in human macrophages. Identification of critical cell signaling pathways regulated by CREB that favor bacterial growth could reveal promising new targets for host-directed therapy for TB.

## Introduction

*Mycobacterium tuberculosis* (*M*.*tb*), the causative agent of tuberculosis (TB), is arguably the oldest known human bacterial pathogen. Great strides have been made in the last century in terms of drug development, public education, and disease prevention, however these efforts have fallen short of their goals. With the emergence of the COVID-19 pandemic, limited access to life saving diagnostics, drug treatment and preventative measures have led to an increased number of TB deaths from 2019 to 2020, the first rise in over a decade [1]. Incidence of infection with drug-resistant *M*.*tb* continues to be a challenge, demonstrating the urgent need for novel therapeutic approaches to combat TB.

Resident alveolar macrophages (AMs) are the first cells to encounter *M*.*tb* after inhalation into the lungs [2,3], and the initial interactions between the bacteria and AMs can dictate the outcome of infection [4–6]. During pathogenesis, *M*.*tb* disseminates through the body, coming into contact with tissue macrophages that are mostly monocyte-derived [7]. *M*.*tb* is known to hijack macrophage processes to create an environment conducive to bacterial growth. These include prevention of phagosome maturation and fusion with lysosomes, interference in host signaling pathways such as inhibition of nuclear factor kappa B (NF-kB) signaling, modulation of programed cell death pathways, and others [8]. It is critical that we understand the mechanisms of immune evasion employed by the bacteria in order to identify new targets in host cells to enhance host defense against *M*.*tb* in addition to antibiotics through host-directed therapy (HDT).

One of our goals is to identify the intracellular “master regulators” and inflammatory metabolites that dictate human macrophage immunologic responses to *M*.*tb* infection. cAMP Response Element Binding Protein (CREB) is a transcription factor with critical roles in cell survival, proliferation and differentiation [9] and is becoming more appreciated for its important roles in immune function [10]. Upon activation, CREB is phosphorylated on serine 133, translocates to the nucleus, recruits the CREB binding protein (CBP)/p300, and binds to the cyclic adenosine monophosphate (cAMP) regulatory element (CRE) in target gene promotors to induce a transcriptional program generally associated with promoting anti-inflammatory, immunoregulatory cellular responses [10,11]. Previous work in murine macrophage-like cell lines and bone marrow-derived macrophages (BMDMs) has shown that infection with *M*.*tb* and other mycobacteria induce phosphorylation of CREB (pCREB) [12,13]. Further, CREB siRNA-mediated knockdown in the RAW murine macrophage-like cell line resulted in decreased *M*.*tb* growth [14]. However, the effector functions of CREB in human macrophages infected with *M*.*tb* are virtually unknown.

In the current study, we sought to investigate the role of CREB during *M*.*tb* infection of human macrophages. Using human monocyte-derived macrophages (MDMs), we determined that infection with *M*.*tb* induces CREB phosphorylation independent of cAMP and is dependent on the p38/MAPK pathway. We also observed that CREB is important for induction of certain immediate early genes, including COX2, MCL-1, CCL8 and c-FOS. Further, we show that inhibition of CREB results in increased nuclear localization of NF-kB and decreased intramacrophage *M*.*tb* growth. Finally, we determined that CREB is important for limiting activation of RIPK3/MLKL unaccompanied by necroptotic cell death. Instead, we show that CREB activation limits intracellular trafficking of *M*.*tb* to the phagolysosome through inhibition of MLKL phosphorylation, a novel effector function for CREB in this context. Altogether, we show that CREB activation acts as a critical mechanism by which *M*.*tb* evades the immune system in human macrophages and may represent a viable target pathway for development of HDTs.

## Results

### *M*.*tb* infection induces CREB phosphorylation in human macrophages independent of cAMP production

**Fig 1:**
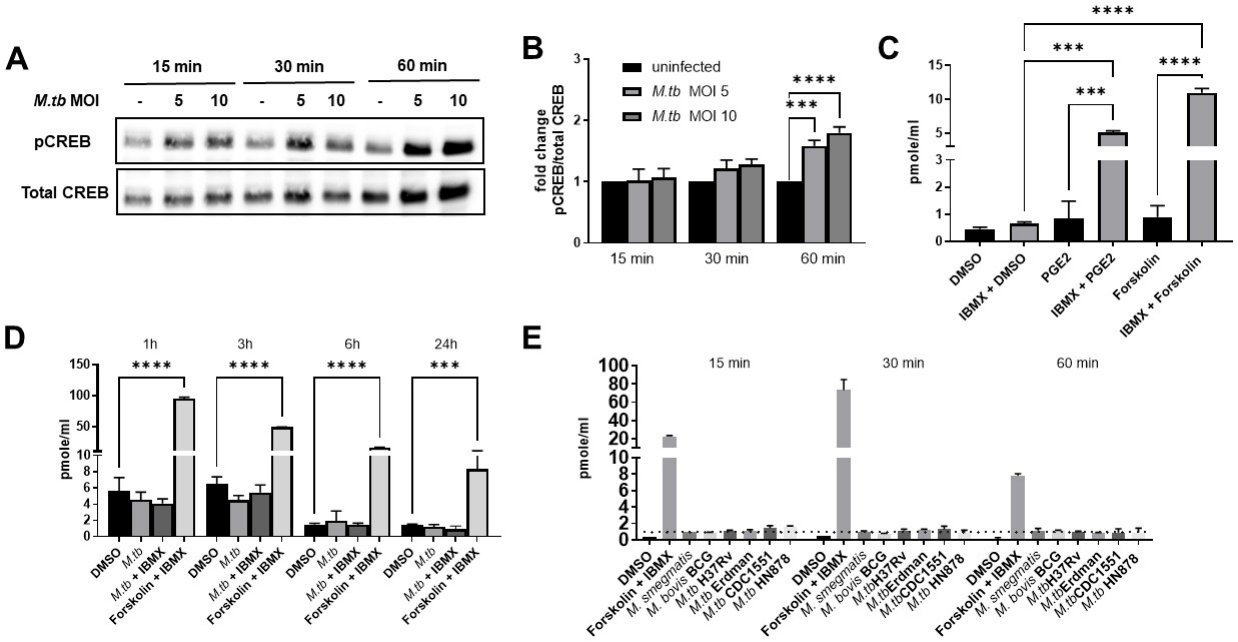
*M*.*tb* induces CREB activation independent of cAMP in human macrophages. MDMs were infected with *M*.*tb* H_37_R_v_ by synchronized phagocytosis at the indicated MOI. A) Western blot was performed to detect levels of phosphorylated and total CREB protein at the indicated time points. WB is representative of n = 4 donors. B) Densitometry analysis was performed and ratios of pCREB/total CREB were determined. Data are cumulative ± SEM of n = 4 donors. One-way ANOVA with Tukey’s post-test. C) MDMs were stimulated with cAMP agonists PGE_2_ or forskolin ± PDE inhibitor IBMX for 30 min. Data are representative ± SD of n = 4 donors. One-way ANOVA with Tukey’s post-test. D) cAMP levels in *M*.*tb*-infected (MOI 5) MDM lysates were determined. Graph is representative ± SD of n = 11 donors. One-way ANOVA with Tukey’s post-test. E) MDMs were infected with different strains of mycobacteria (MOI 5) or forskolin + IBMX as a control for cAMP production. Lysates were collected and analyzed for cAMP production. Dotted line indicates minimum level of detection for the assay. Data are representative ± SD of n = 2 donors; *p < 0.05, ***p < 0.001, ****p < 0.0001.

Previous work in J774.1 murine macrophage-like cells, THP-1 cells and in vivo mouse models showed that infection of macrophages with mycobacteria induced CREB phosphorylation in a manner dependent on cAMP signaling, and additionally that CREB is important for bacterial pathogenesis [12]. We sought to determine the translatability of these findings to humans using an MDM model of *M*.*tb* infection. We infected MDMs with virulent *M*.*tb* strain H_37_R_v_ and confirmed that *M*.*tb* infection induces CREB phosphorylation in primary human macrophages (Fig 1A). Densitometry showed that by 60 min post infection, pCREB was significantly increased over baseline levels (Fig 1B). To determine if phosphorylation of CREB was due to cAMP in human macrophages, we first wanted to establish the capability of MDMs to produce measurable levels of cAMP. We stimulated the MDMs with PGE_2_ or forskolin, known agonists of adenylate cyclase that converts ATP to cAMP. We also added IBMX, a pan-inhibitor of phosphodiesterases that quickly degrade cAMP, in order to maximize our ability to detect cAMP. Both PGE_2_ and forskolin, when used with IBMX, induced significantly increased levels of cAMP in MDMs (Fig 1C). We then infected MDMs with *M*.*tb* alone or in combination with IBMX treatment. We did not detect *M*.*tb*-induced cAMP at the time points tested, however cAMP was plentiful in the positive control wells (Fig 1D). In addition, we stimulated MDMs with various strains of *M*.*tb*, *M*. *smegmatis* and *M*. *bovis* BCG at early time points and did not observe significantly elevated levels of cAMP elicited by mycobacterial infection (Fig 1E). These data indicate that *M*.*tb* infection of human macrophages induces activation of CREB early post-infection independent of cAMP production.

### *M*.*tb*-induced CREB phosphorylation in human macrophages is dependent on p38 MAPK

**Fig 2:**
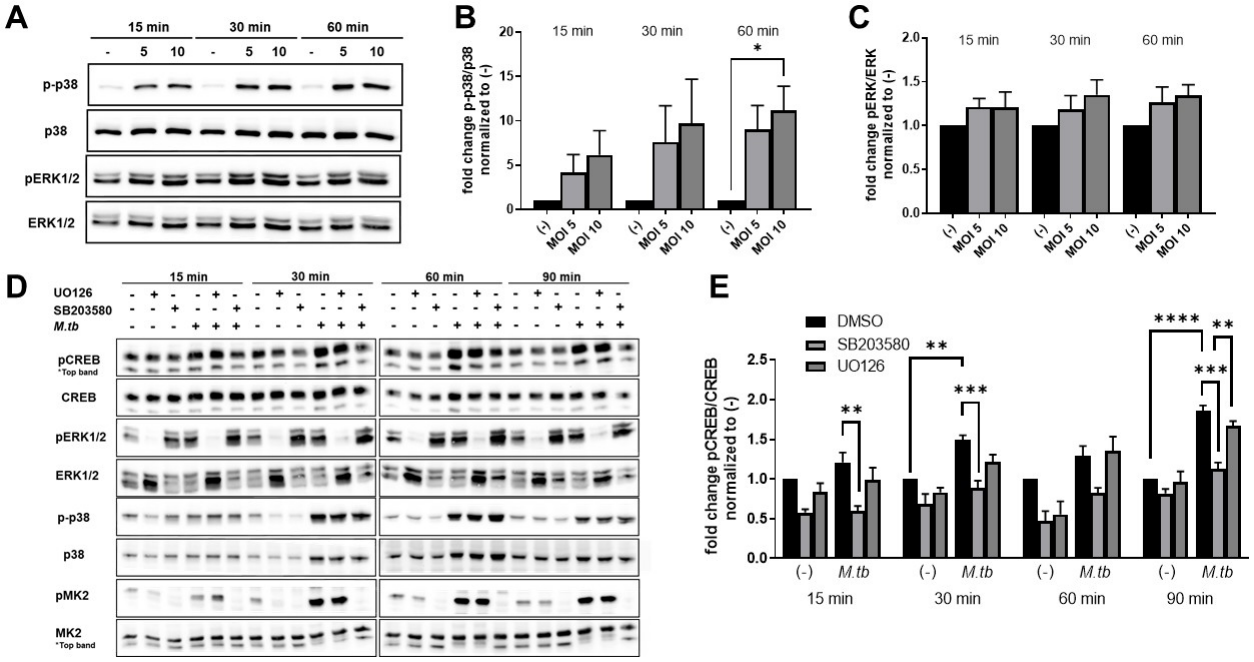
*M*.*tb*-induced CREB activation is p38-dependent and ERK-independent. A) MDMs were infected with *M*.*tb* H_37_R_v_ by synchronized phagocytosis at the indicated MOI. WB was performed to detect levels of phosphorylated and total p38 and ERK1/2 protein at the time points specified. Data are representative of n = 4 donors. B,C) Densitometry of phosphorylated protein normalized to total protein. Data are cumulative ± SEM of n = 4 donors. One-way ANOVA with Tukey’s post-test. D-E) MDMs were pretreated with SB203580 or UO126 for 60 min prior to infection with *M*.*tb* H_37_R_v_ at MOI 10. D) WB was performed to detect levels of phosphorylated and total levels of CREB, p38, ERK1/2, and MK2 protein at the specified time points. Data are representative of n = 4 donors. E) Densitometry of phosphorylated protein normalized to total protein. Data are cumulative ± SEM of n = 4 donors. One-way ANOVA with Tukey’s post-test; *p < 0.05, **p < 0.01, ***p < 0.001, ****p < 0.0001.

In light of our data showing no change in cAMP levels following *M*.*tb* infection, we sought to identify the pathway used by *M*.*tb* to induce phosphorylation of CREB in human macrophages. The mitogen activated protein kinase (MAPK) pathway is another common signaling pathway associated with CREB activation [10]. Both ERK1/2 and p38 are upstream activators of CREB signaling in macrophages [13,15]. Along with other groups, we have previously shown that *M*.*tb* infection of human macrophages induces phosphorylation of MAPK p38 [16], suggesting that this is a potential pathway for *M*.*tb* induction of pCREB. We found that *M*.*tb* infection induced p-p38 as early as 15 min post-infection with significantly more p-p38 at 1h post infection (Fig 2A and 2B). We also investigated phosphorylation of ERK1/2 by *M*.*tb* and detected a slight increase in phosphorylation, which was not significantly different compared to uninfected macrophages (Fig 2A and 2C). To determine if p38 or ERK1/2 signaling is required for CREB phosphorylation, we pretreated MDMs with inhibitors of p38 or ERK1/2 (Fig 2D). UO126 inhibited phosphorylation of ERK1/2 and this was confirmed by WB (Fig 2D). SB203580 does not inhibit phosphorylation of p38, but inhibits its kinase activity. We confirmed the inhibitor’s effect by probing for phosphorylated MK2, a downstream target of p38, by Western blot (Fig 2D). We observed that inhibition of p38, but not ERK1/2 resulted in a loss of *M*.*tb*-induced CREB phosphorylation (Fig 2D and 2E), with pCREB levels in infected cells treated with SB203580 remaining similar to baseline levels at all time points tested. Thus, we determined that p38 signaling is critical for CREB activation by *M*.*tb* in human macrophages.

### CREB activation regulates induction of immediate early genes in *M*.*tb*-infected human macrophages

Having identified the signaling pathway required for *M*.*tb*-induced CREB phosphorylation, the next critical issue was to identify the effector functions of CREB in *M*.*tb*-infected macrophages. To investigate CREB’s role(s) in *M*.*tb* pathogenesis, we employed the CREB-specific inhibitor, 666–15. 666–15 has been shown to inhibit the interaction between CREB and the CREB-binding protein (CBP) [17], a transcription coactivator critical for formation of CREB’s transcription complex [10,11]. Previous reports indicated that 666–15 does not inhibit phosphorylation of CREB protein, however, we were curious whether this inhibitor impeded CREB translocation to the nucleus, a required step in CREB’s transcriptional activity. We observed nuclear localization of pCREB by confocal microscopy and quantified the fluorescent signal that overlapped with the nuclear stain DAPI (S1A and S1B Fig). *M*.*tb* infection of MDMs resulted in a significant increase in pCREB nuclear translocation at 1h post-infection compared to uninfected cells. Treatment with 666–15 resulted in decreased nuclear pCREB signal compared to infected cells treated with DMSO. To rule out the possibility that 666–15 alters bacterial association with MDMs and thus potentially confound the data, we quantified association using confocal microscopy. We did not detect a difference in bacterial association with MDMs (S1C Fig). The reduction in nuclear localization of pCREB in human macrophages demonstrates a newly described mechanism for 666–15 in the inhibition of CREB activity.

**Fig 3:**
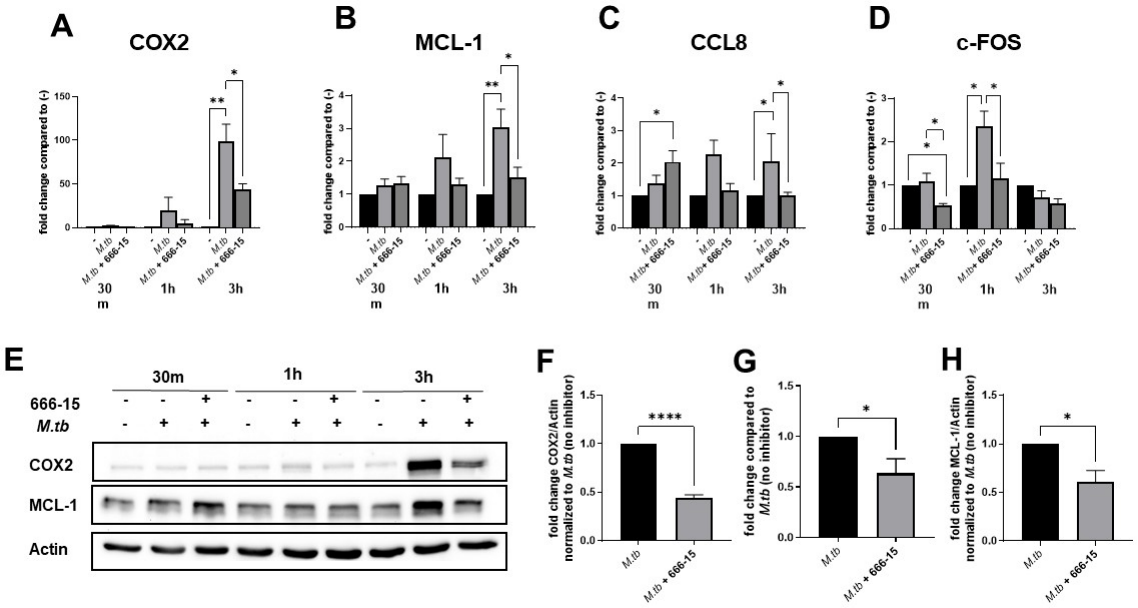
*M*.*tb*-induced CREB activation is important for induction of immediate early genes in human macrophages. MDMs were pretreated with DMSO or 666–15 for 60 min and subsequently infected with *M*.*tb* H_37_R_v_ at the MOI 10 (A-F,H) by synchronized phagocytosis or MOI 5(E). RNA or protein lysates were collected at the indicated time points. A-D) Gene expression of COX2, MCL-1, CCL8 and c-FOS was determined by qRT-PCR and data are shown as fold change ± SEM compared to uninfected MDMs. Data are cumulative of n = 3–5 donors. One-way ANOVA with Tukey’s post-test. E) Cell lysates were probed by WB for COX2, MCL-1 and β-actin. A representative experiment is shown of n = 3–4 donors. F,H) Densitometry at 3h post infection compared to *M*.*tb*-infected MDMs. Data are cumulative ± SEM of n = 3–4 donors. Unpaired t test. G) PGE_2_ production was measured by competitive binding ELISA at 6h post infection. Data are cumulative ± SEM of n = 4 donors. Unpaired t test; *p < 0.05, **p < 0.01, ****p < 0.0001.

One of CREB’s vital roles as a transcription factor and master regulator of anti-inflammatory immune responses is to rapidly induce transcription of immediate early genes (IEGs). These genes include cytokines, chemokines, growth factors, transcription factors and nuclear receptors [9,10]. We first sought to determine the role of *M*.*tb*-induced CREB activation in regulation of select IEGs identified in macrophage literature and confirmed via GeneHancer to have predicted CREB binding sites (Fig 3A–3D, S2A Fig) [9,10,14,18–24]. We investigated gene expression over a time course of 30 min to 3h post-infection, considering that transcription levels of many IEGs will peak early and may return to baseline thereafter. *M*.*tb* infection increased gene expression levels of several IEGs including cyclooxygenase 2 (COX2), myeloid cell leukemia-1 (MCL-1), chemokine (C-C motif) ligand 8 (CCL8), and FOS proto-oncogene (c-FOS) in a CREB-dependent manner in this time frame albeit at different time points (Fig 3A–3D, S2A Fig). Whereas CREB inhibition abrogated the enhanced expression levels of MCL-1 and CCL8 due to *M*.*tb* infection at 1 and 3h, CREB inhibition did not completely abrogate COX2 gene expression at 3h, indicating the possibility that a secondary signaling pathway is activated by *M*.*tb* infection independent of CREB.

Interestingly, transcription levels of CCL8 increased with CREB inhibition in *M*.*tb*-infected MDMs at 30 min compared to control, suggesting that CREB may have an early, inhibitory effect on CCL8 transcription (Fig 3C). However, over time, *M*.*tb* infection resulted in significantly increased CCL8 transcript levels, which were abrogated with CREB inhibition. CREB was also critical for induction of c-FOS transcription (Fig 3D). Inhibition of CREB reduced transcript levels below baseline at 30 min post-infection, and abolished *M*.*tb*-induced c-FOS levels which peaked at 1h post-infection. Of note, we also detected decreased protein production for c-FOS in CREB inhibited, *M*.*tb* infected macrophages at 3h post-infection (S2B and S2C Fig). Additional genes were investigated with three outcomes for the majority of genes. Either *M*.*tb* infection did not alter transcript levels, inhibition of CREB did not significantly affect *M*.*tb*-induced changes in gene expression, or initial effects of CREB inhibition were transient with no significant effect by 3h post-infection (S2A Fig).

Previous studies have determined that COX2 and MCL-1 are induced by *M*.*tb* infection in human macrophages [16,25,26]. COX2 is responsible for production of the eicosanoid prostaglandin E_2_ (PGE_2_), which can limit *M*.*tb* growth in mouse models [27,28], however, PGE_2_’s role in macrophages early post-infection is less clear. MCL-1 is critical for regulation of apoptosis and previously shown by our lab and others to be critical for *M*.*tb* growth in human macrophages [25,29]. Considering that inhibition of CREB led to decreased gene expression for COX2 and MCL-1, we next sought to determine if there was an effect on protein production for these IEGs. At 3h post-infection, we observed increased COX2 and MCL-1 protein levels in *M*.*tb*-infected compared to uninfected MDMs, and this increase was abrogated with CREB inhibition (Fig 3E). Densitometry analysis revealed that COX2 protein production was significantly decreased in CREB-inhibited *M*.*tb*-infected MDMs compared to *M*.*tb*-infected MDMs with intact CREB signaling at 3h (Fig 3F). Consistent with this, we detected decreased levels of *M*.*tb*-induced PGE_2_ production at 6h post-infection in CREB-inhibited MDMs compared to control (Fig 3G). There was also significantly less MCL-1 protein expression in CREB inhibited, *M*.*tb*-infected MDMs (Fig 3H), following the pattern observed with MCL-1 gene expression. Altogether, these data reveal that CREB signaling is important for *M*.*tb*-induced gene and protein expression of COX2 and MCL-1 early post-infection in human macrophages, as well as PGE_2_ production. Considering the important roles that COX2, PGE_2_ and MCL-1 play in *M*.*tb* pathogenesis [25–29], these results have important implications for CREB’s role in early mechanisms of immune evasion by *M*.*tb*.

### *M*.*tb*-induced CREB activity limits NF-kB nuclear localization and allows for bacterial growth in human macrophages

**Fig 4:**
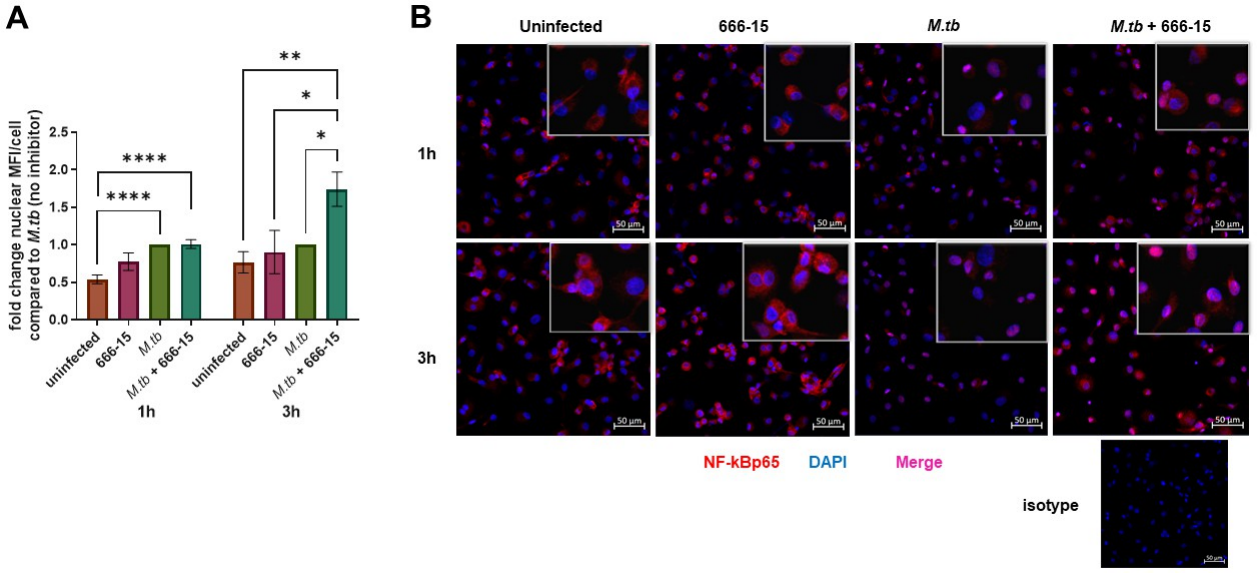
Inhibition of *M*.*tb*-induced CREB activation allows for prolonged nuclear colocalization of NF-kB p65 in human macrophages. MDMs were pretreated with DMSO or the CREB inhibitor, 666–15, for 60 min then infected with *M*.*tb* H_37_R_v_ at MOI 10. A) At 1h and 3h post-infection, cells were fixed, permeabilized, and stained for NF-kB p65 (red) and DAPI in the nucleus (blue). MFI of NF-kB p65 signal that colocalized with DAPI (magenta) was calculated using ImageJ software, normalized to total number of cells per field and graphed as fold change ± SEM compared to infected cells. Data are cumulative of n = 3–6 donors. One-way ANOVA with Tukey’s post-test. B) Images were taken at 3h post-infection at 20x magnification. Images shown are representative of n = 3–6 donors; *p < 0.05, **p < 0.01, ****p < 0.0001.

The transcription factor nuclear factor kappa B (NF-kB) is largely responsible for inducing transcription of pro-inflammatory genes, generating an antimicrobial environment [30,31]. NF-kB activity is important for host protection against *M*.*tb* [32–34]. Previous reports show that CREB signaling can hinder pro-inflammatory signaling pathways regulated by NF-kB in part because CREB and NF-kB compete in the nucleus for binding to the CREB binding protein CBP/p300 [10,35]. Both CREB and NF-kB require CBP/p300 to form their respective transcription complexes [10]. We determined that inhibition of CREB with 666–15 resulted in decreased nuclear localization of pCREB (S1 Fig), therefore, we next investigated whether or not this decrease resulted in increased NF-kB nuclear localization. We observed that *M*.*tb* infection significantly induced NF-kB nuclear colocalization at 1h post infection compared to uninfected controls which show NF-kB localizing predominantly in the cytoplasm (Fig 4A and 4B). Inhibition of CREB with 666–15 during *M*.*tb* infection had no significant effect on NF-kB localization compared to *M*.*tb* infection alone at 1h. However, by 3h post-infection, nuclear localization of NF-kB in the *M*.*tb*-infected, CREB-inhibited MDMs was significantly increased compared to uninfected as well as *M*.*tb*-infected cells (Fig 4A and 4B). Furthermore, treatment with the CREB inhibitor without *M*.*tb* infection was insufficient for nuclear localization of NF-kB at both time points tested (Fig 4A). These data indicate that *M*.*tb*-induced CREB activation inhibits prolonged nuclear localization of NF-kB in human macrophages, which is expected to negatively impact host response to *M*.*tb*.

**Fig 5:**
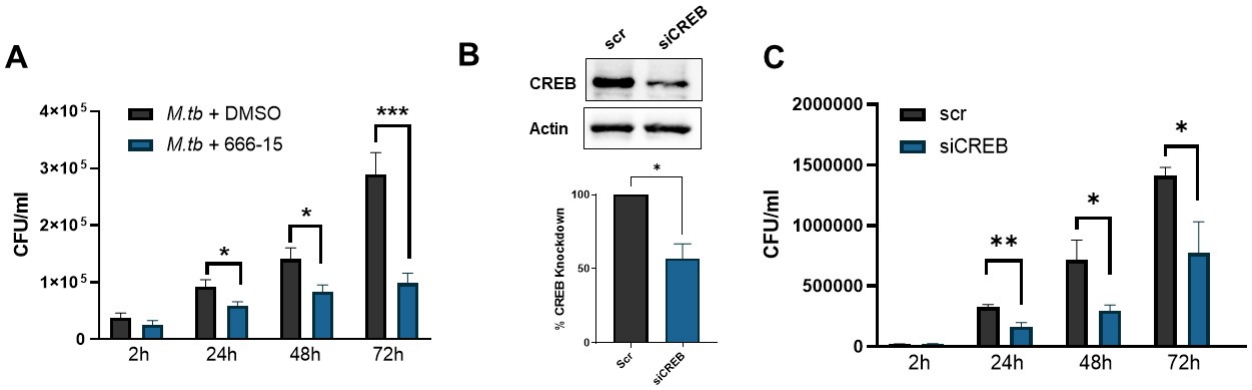
*M*.*tb*-induced CREB activation is important for bacterial growth in human macrophages. A) MDMs were pretreated for 60 min with CREB inhibitor 666–15 or DMSO, then infected with *M*.*tb* H_37_Rv MOI 2 and CFUs were determined at the indicated time points. Data are cumulative ± SEM of n = 6 donors. Unpaired t test. B) MDMs were transfected with CREB targeting siRNA or scrambled control for 72h prior to infection with *M*.*tb* H_37_R_v_ MOI 2. A representative WB is shown and graphed data are cumulative ± SEM of n = 2 donors. C) CFUs were determined at the indicated time points. Data are representative ± SD of n = 2 donors. Unpaired t test; *p < 0.05, **p < 0.01, ***p < 0.001.

Altogether, the early CREB-mediated signaling events predicted that CREB inhibition would lead to enhanced macrophage control of *M*.*tb* growth. To test this, bacterial burden in MDMs treated with the CREB inhibitor was determined at 2, 24, 48 and 72h post infection by CFU assay (Fig 5A). To preserve the MDM monolayer for these extended time points, an MOI of 2 was used and confirmed to significantly increase pCREB (S3A and S3B Fig). At 2h post infection, the level of infection was not significantly different, indicating that the inhibitor did not have an effect on *M*.*tb* phagocytosis by MDMs, confirming our previous observation (S1C Fig). We observed that *M*.*tb* growth increased over time, as expected. Remarkably, CREB inhibition by 666–15 significantly reduced the bacterial burden in treated macrophages compared to DMSO-treated control macrophages as early as 24h post-infection through the end of the assay at 72h (Fig 5A). To validate these results, we used siRNA to knock down CREB prior to infection with *M*.*tb* (Fig 5B), and observed that CREB deficient macrophages were better able to control *M*.*tb* growth through the time points tested (Fig 5C). Together, these data clearly show the important role of CREB in bacterial growth in human macrophages.

### CREB activation negatively regulates the necroptotic signaling pathway

**Fig 6:**
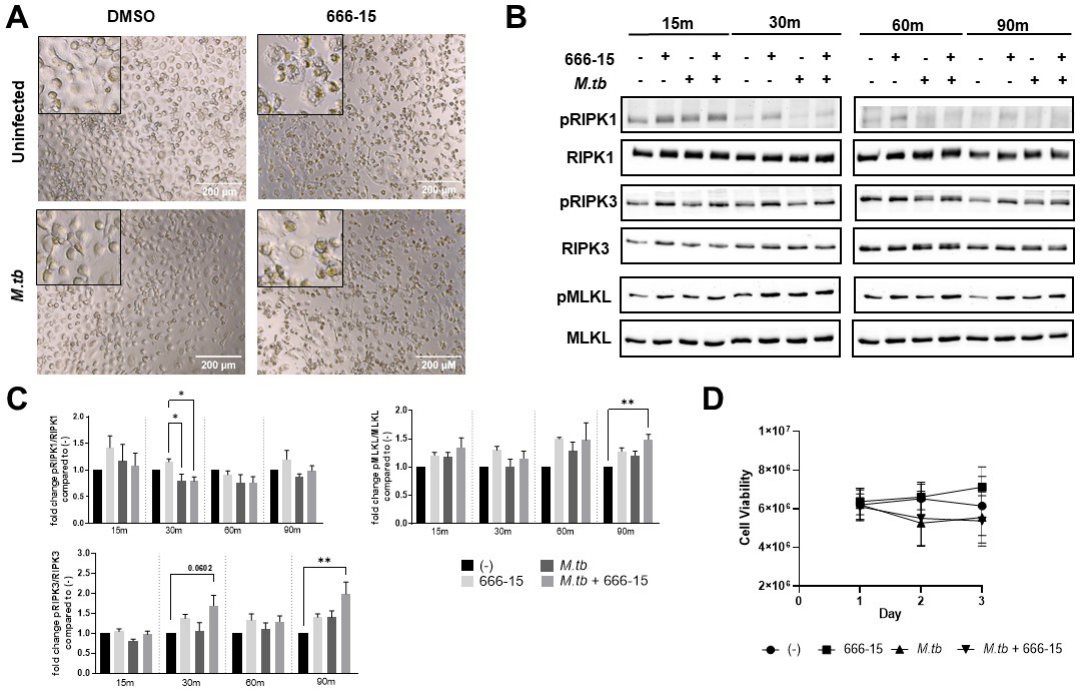
CREB inhibition induces activation of the necroptotic signaling pathway in *M*.*tb*-infected macrophages, but does not affect cell viability. MDMs were pretreated with 666–15 or DMSO control for 60 min and subsequently infected with *M*.*tb* H_37_R_v_ at MOI 2 (A,D) or MOI 10 (B,C). A) Bright field images of the cells were taken at 40x; Data shown are representative of n = 10 donors. B,C) Cell lysates were collected and probed by WB blot for the indicated phosphorylated and total proteins. Densitometry was determined and graphed as fold change ± SEM of phosphorylated protein to total protein; A representative experiment is shown and graphed data are cumulative of n = 4 donors. One-way ANOVA with Tukey’s post-test. D) Membrane integrity and cell viability was determined by Cytotox Glo assay. Data are cumulative ± SEM of n = 3 donors. Two-way ANOVA with Tukey’s post-test; *p < 0.05, **p < 0.01, ***p < 0.001.

During the course of the CFU experiments, we observed morphological changes in the 666–15 treated cells at the extended time points. MDMs treated with 666–15, regardless of infection status, began to exhibit subtle cellular swelling or blebbing as early as 24h post-treatment, peaking at 72h post-treatment (Fig 6A). This morphology was not discerned in the uninfected or *M*.*tb*-infected MDMs treated with DMSO. Based on what appeared to be cellular swelling and a recent report suggesting CREB negatively regulates gene expression of necroptotic signaling pathway genes in neurons [36,37], we hypothesized that the CREB inhibitor was inducing necroptosis in the MDMs. The necroptotic signaling pathway is initiated by phosphorylation of receptor-interacting protein kinase 1 (RIPK1) and RIPK3, resulting in phosphorylation of mixed lineage kinase domain like pseudokinase (MLKL). Phosphorylated MLKL will then oligomerize and accumulate on the cellular membrane, forming pores and releasing damage-associated molecular patterns (DAMPs). When accumulation of MLKL reaches a breaking point, the cell will lyse releasing inflammatory DAMPs. We probed cell lysates for phosphorylated RIPK1, RIPK3 and MLKL by Western blot over a time course from 15–90 min post-infection (Fig 6B). Band density was quantified and normalized to total protein (Fig 6C). We detected a trend towards increased pRIPK1 in 666–15 treated, uninfected MDMs as early as 15 min post infection, although not significantly increased compared to resting cells. *M*.*tb* infection did not stimulate phosphorylation of RIPK1 at any time point tested, but did decrease the phosphorylation elicited by the CREB inhibitor at 30 min post-infection, suggesting that *M*.*tb* may transiently inhibit early activation of RIPK1 in a CREB-independent manner.

RIPK3 is downstream of RIPK1, but is also activated independently of RIPK1. We observed trends toward increased RIPK3 phosphorylation with 666–15 treatment in uninfected cells beginning at 30 min through 90 min post-treatment, though not significantly increased compared to resting cells (Fig 6C). However, there was an additive effect in *M*.*tb*-infected, 666-15-treated MDMs beginning at 30 min post-infection which culminated in significantly increased pRIPK3 at 90 min post-infection compared to resting cells. We ascertained similar findings for MLKL phosphorylation, including the significant additive effect in *M*.*tb*-infected, 666-15-treated MDMs at 90 min post infection (Fig 6C).

In light of these data showing activation of the necroptotic signaling pathway, we next conducted assays to determine cell viability and deduce the occurrence of necroptosis due to phosphorylation of MLKL. We used a cell viability assay that measures cell death based on cell membrane rupture, which occurs during necroptosis. To our surprise, we did not detect a significant difference in viability of MDMs treated with 666–15 compared to DMSO treated cells in both *M*.*tb*-infected and uninfected macrophages (Fig 6D). We saw similar results by lactate dehydrogenase (LDH) release assay (S4 Fig). We concluded that while the necroptotic signaling pathway is activated by inhibiting CREB during *M*.*tb* infection, necroptotic cell death is not occurring. Non-necroptotic cell death roles for this signaling pathway have been recently identified [36]. Our data suggest that CREB appears to regulate a function for MLKL that does not include the induction of cell death over the time period tested.

**Fig 7:**
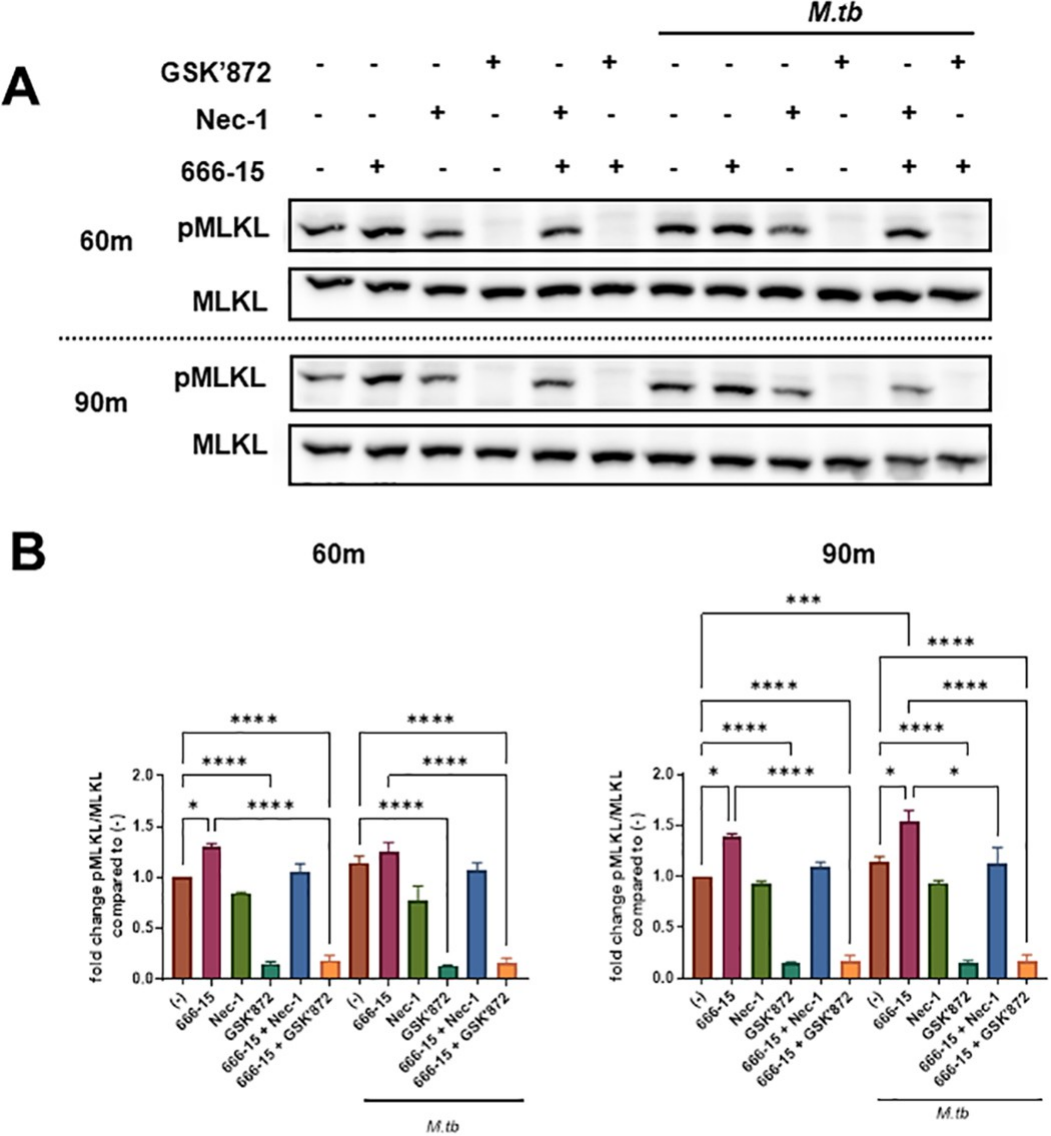
pMLKL induced by CREB inhibition in human macrophages infected with *M*.*tb* is dependent on RIPK1/RIPK3. A) MDMs were pretreated with for 60 min with DMSO or CREB inhibitor 666–15 +/- Nec-1 or GSK’872 then infected or not with *M*.*tb* H_37_R_v_ MOI 10. Cell lysates were collected and probed by WB for phosphorylated and total MLKL at the indicated time points. A representative experiment is shown of n = 5, 4 donors. B) Densitometry was determined and graphed as fold change ± SEM of phosphorylated protein to total protein. n = 5, 4 donors. One-way ANOVA with Tukey’s post-test; *p < 0.05, **p < 0.01, ***p < 0.001, ****p < 0.0001.

Considering that we did not detect cell death, we wanted to better understand the mechanism(s) for MLKL phosphorylation in the *M*.*tb*-infected, CREB inhibited macrophages. MLKL activation is largely dependent on RIPK3 and can occur independent of RIPK1 signaling. To determine whether RIPK1 or RIPK3 or both were required for pMLKL in response to CREB inhibition ± *M*.*tb* infection, we employed inhibitors of RIPK1 (necrostatin, Nec-1) and RIPK3 (GSK’872). Nec-1 did not significantly alter baseline levels of pMLKL (Fig 7A and 7B). RIPK1 inhibition by Nec-1 also did not significantly affect pMLKL induced by 666–15 alone. However, at 60 and 90 min post-infection with *M*.*tb*, Nec-1 treatment resulted in a 14.99 ± 8.03% (N = 5) and 28.47±12.64% (N = 5) decrease in pMLKL, respectively, compared to *M*.*tb* infected/CREB inhibited macrophages (maroon versus blue bars), reaching significance at 90 min. On the other hand, inhibition of RIPK3 by GSK’872 markedly inhibited both baseline levels of pMLKL compared to uninfected controls and the significant increase in pMLKL induced by CREB inhibition ± *M*.*tb* infection (Fig 7). This demonstrates the critical role played by RIPK3 in phosphorylation of MLKL in human macrophages both constitutively and in response to infection and a smaller role for RIPK1 in response to infection. These data are the first to show that CREB negatively regulates phosphorylation of RIPK1, RIPK3 and MLKL, and that *M*.*tb*-induced CREB activation inhibits phosphorylation of RIPK3/MLKL (Fig 6), coinciding with *M*.*tb* growth in human macrophages.

### Trafficking of *M*.*tb* in human macrophages is dependent on RIPK3-induced pMLKL

**Fig 8:**
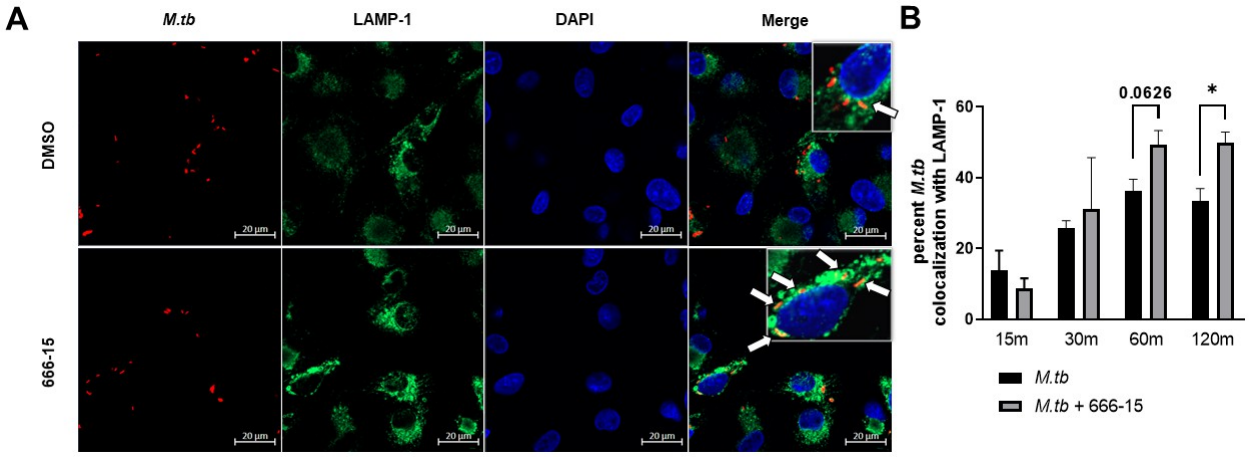
CREB signaling in human macrophages inhibits phagolysosomal fusion. A) MDMs were plated on glass coverslips and pretreated with DMSO or CREB inhibitor 666–15 for 60 min then infected with mCherry expressing *M*.*tb* H_37_R_v_ (red) MOI 10. MDMs were fixed, permeabilized, and stained for LAMP-1 (green) and DAPI (blue). A representative experiment is shown of n = 3 donors. B) At the indicated time points, the percent of *M*.*tb* colocalizing with LAMP-1 was calculated following manual counting. White arrows indicate colocalization. Data are cumulative ± SEM of n = 3 donors. Unpaired t test; *p < 0.05.

Additional functions beyond necroptosis have been reported for RIPK3 and MLKL [36], including a recent report indicating that pMLKL can be targeted for endosomal degradation which can prompt enhanced targeting of *Listeria monocytogenes* and *Yersinia enterocolitica* to lysosomes [38]. It is well known that *M*.*tb* inhibits phagosome maturation and fusion with lysosomes, allowing for bacterial survival [39–41]. Our data show that CREB inhibition leads to increased phosphorylation of MLKL, therefore, we next investigated the role of CREB in bacterial trafficking, an unexplored area of CREB signaling. We inhibited CREB signaling in MDMs and infected the macrophages with mCherry-expressing *M*.*tb* H_37_R_v_. At the indicated time points, we quantified colocalization of bacteria with lysosome associated membrane protein-1 (LAMP-1), indicating fusion of the endosome with late endosomes or lysosomes (Fig 8A and 8B). We detected significantly increased colocalization of LAMP-1 with *M*.*tb* in CREB-inhibited MDMs compared to control MDMs at 2h post-infection (Fig 8B). These data indicate a novel role for CREB in regulation of phagosome maturation and phagolysosomal fusion during *M*.*tb* infection in human macrophages.

**Fig 9:**
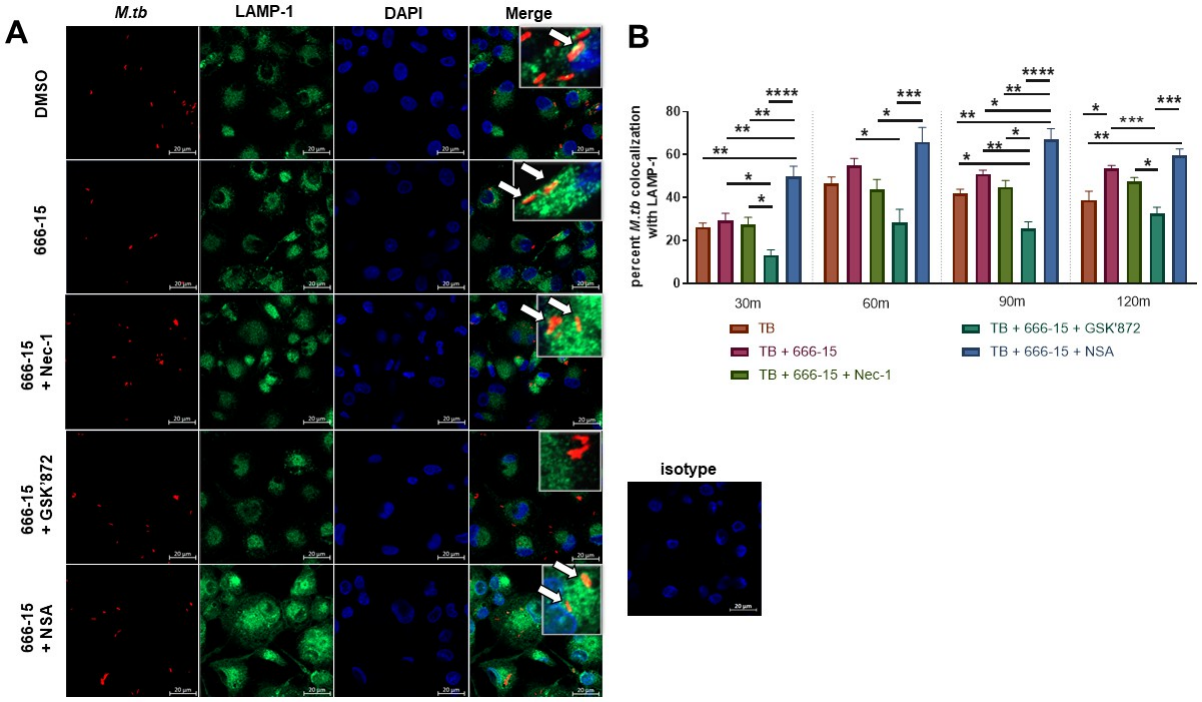
Increased phagolysosomal fusion during CREB inhibition requires RIPK3 activity. A) MDMs were plated on glass coverslips and pretreated for 60 min with DMSO or CREB inhibitor 666–15 +/- Nec-1, GSK’872, or NSA, then infected with mCherry *M*.*tb* H_37_R_v_ (red) MOI 10. MDMs were fixed, permeabilized, and stained for LAMP-1 (green) and DAPI (blue). A representative experiment is shown of n = 4–5 donors. B) At the indicated time points, the percent of *M*.*tb* colocalizing with LAMP-1 was calculated following manual counting. White arrows indicate colocalization. Data are cumulative ± SEM of n = 4–5 donors. One-way ANOVA with Tukey’s post-test; *p < 0.05, **p < 0.01, ***p < 0.001, ****p < 0.0001.

To determine whether the enhanced phagolysosome fusion detected during CREB inhibition is due to activation of the necroptotic signaling pathway, we pretreated MDMs with CREB inhibitor 666–15 +/- Nec-1 (RIPK1 inhibitor), GSK’872 (RIPK3 inhibitor) or necrosulfonamide (NSA, MLKL inhibitor) for 1h prior to infection with mCherry expressing *M*.*tb* H_37_R_v_. NSA does not inhibit MLKL phosphorylation or oligomerization, but inhibits MLKL translocation to the cell membrane inhibiting necroptosis [36,42]. At the indicated time points, cells were stained for LAMP-1 (Fig 9A and S5A Fig) and colocalization with fluorescent bacteria was quantified by confocal microscopy (Fig 9B and S5B Fig). We again ascertained that CREB inhibition resulted in increased phagolysosomal fusion by 2h post infection compared to control (Fig 9B and S5B Fig). Addition of Nec-1, GSK’872, or NSA alone did not affect bacterial colocalization with LAMP-1 (S5A and S5B Fig). However, simultaneous inhibition of RIPK3 (GSK ‘872) with CREB (666–15) significantly reduced phagolysosomal fusion compared to CREB-inhibitor only treated MDMs (teal versus maroon bars) at all time points tested (Fig 9B). To investigate MLKL’s role in the increased bacterial trafficking detected during CREB inhibition, we inhibited MLKL with NSA in parallel with CREB inhibition. In contrast to our observations with RIPK3 inhibition, we observed increased phagolysosomal fusion when combining 666–15 with NSA compared to MDMs treated with 666–15 alone (blue versus maroon bars) at 30 and 90 min post-infection. In addition, CREB inhibition paired with NSA resulted in significantly increased phagolysosomal fusion compared to baseline levels (Fig 9B). These data indicate that inhibition of pMLKL translocation to the plasma membrane with NSA is not responsible for the increased *M*.*tb* trafficking induced by CREB inhibition. Recent work showed that phosphorylated MLKL can be targeted to endosomes rather than oligomerizing and moving to the plasma membrane [43]. This shuttling to the endosomes then prompts enhanced trafficking of intracellular bacteria [38,43]. Because NSA does not inhibit phosphorylation of MLKL, the increase in phagolysosomal fusion observed in cells treated with NSA simultaneously with 666–15 implies that phosphorylation of MLKL, not its translocation, is critical for the observed increase in *M*.*tb* trafficking. Further, our data suggest that preventing translocation of pMLKL to the plasma membrane with NSA allows for more trafficking to the endosomes, increasing *M*.*tb* colocalization with LAMP-1.

**Fig 10:**
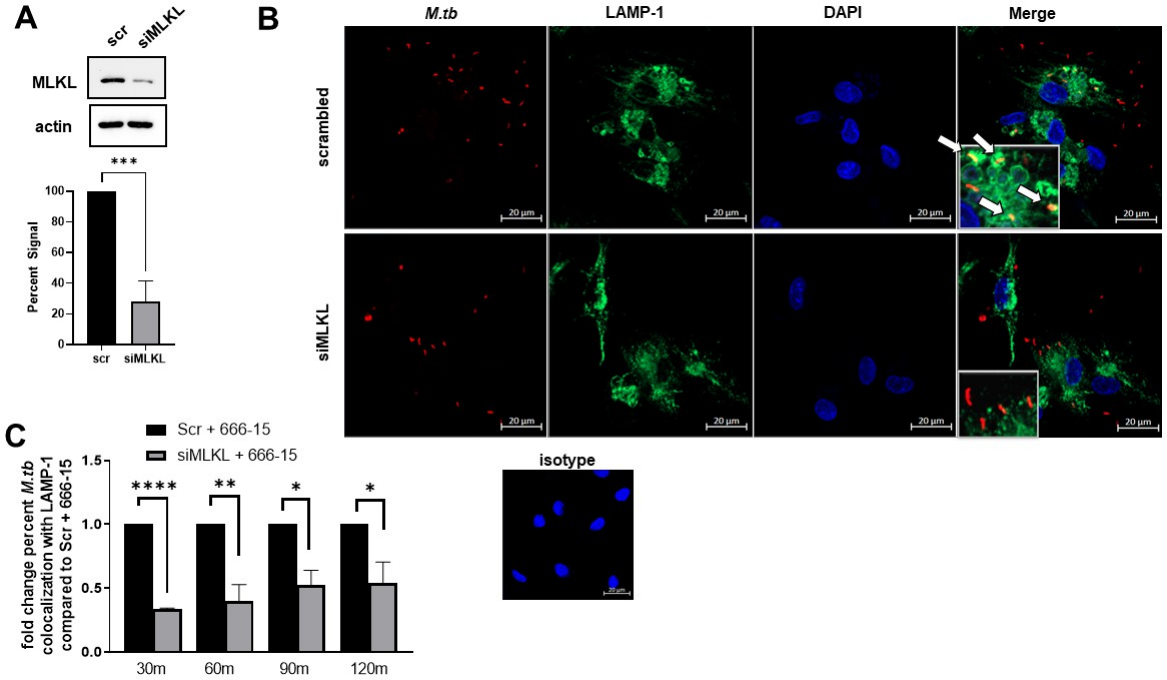
MLKL is essential for phagolysosomal fusion in the absence of CREB signaling in *M*.*tb*-infected human macrophages. MDMs were plated on glass coverslips and transfected with siRNA targeting MLKL or scrambled control siRNA for 48h. MDMs were then pretreated for 60 min with CREB inhibitor 666–15 and infected with mCherry *M*.*tb* H_37_R_v_ (red) MOI 10. A) Knockdown of MLKL was verified by WB and percent signal quantified relative to scrambled control. A representative experiment is shown and graphed data are cumulative of n = 3 donors. Unpaired t test. B) MDMs were fixed, permeabilized, and stained for LAMP-1 (green) and DAPI (blue). A representative experiment is shown of n = 3 donors. C) At the indicated time points, the percent of *M*.*tb* colocalizing with LAMP-1 was calculated following manual counting. Data are shown as fold change of percent colocalization compared to scrambled control for each time point. White arrows indicate colocalization. Data are cumulative ± SEM of n = 3 donors. Unpaired t test; *p < 0.05, **p < 0.01, ***p < 0.001, ****p < 0.0001.

We next sought to address the importance of MLKL phosphorylation induced by RIPK3 activation in the absence of CREB signaling on phagolysosomal fusion. To our knowledge, an inhibitor of MLKL phosphorylation is not commercially available. Therefore, to confirm MLKL’s role in the increased bacterial trafficking detected during CREB inhibition of *M*.*tb*-infected macrophages, we used siRNA to knockdown MLKL protein (Fig 10A) and assessed colocalization of *M*.*tb* with LAMP-1 by confocal microscopy. We detected significantly less phagolysosomal fusion in the siMLKL transfected MDMs compared to those transfected with the scrambled control siRNA in the CREB-inhibited MDMs at all time points tested (Fig 10B and 10C). Our data show that in the absence of *M*.*tb*-induced CREB signaling, inhibition of RIPK3 abolished MLKL phosphorylation (Fig 7) and significantly restricted phagolysosomal fusion (Fig 9). Taken together with the data showing MLKL is required for the increased phagosome-lysosome fusion observed during CREB inhibition (Fig 10), we conclude that phosphorylation of MLKL is essential for increased trafficking of *M*.*tb* to the lysosomes in human macrophages with CREB-deficient signaling. Conversely, CREB signaling leads to blockage of phosphorylated MLKL, limiting fusion of *M*.*tb*-containing phagosomes with lysosomes, corresponding with bacterial growth. Thus, these data reveal a new effector function for CREB in evasion of human macrophage immune responses by *M*.*tb*.

## Discussion

**Fig 11:**
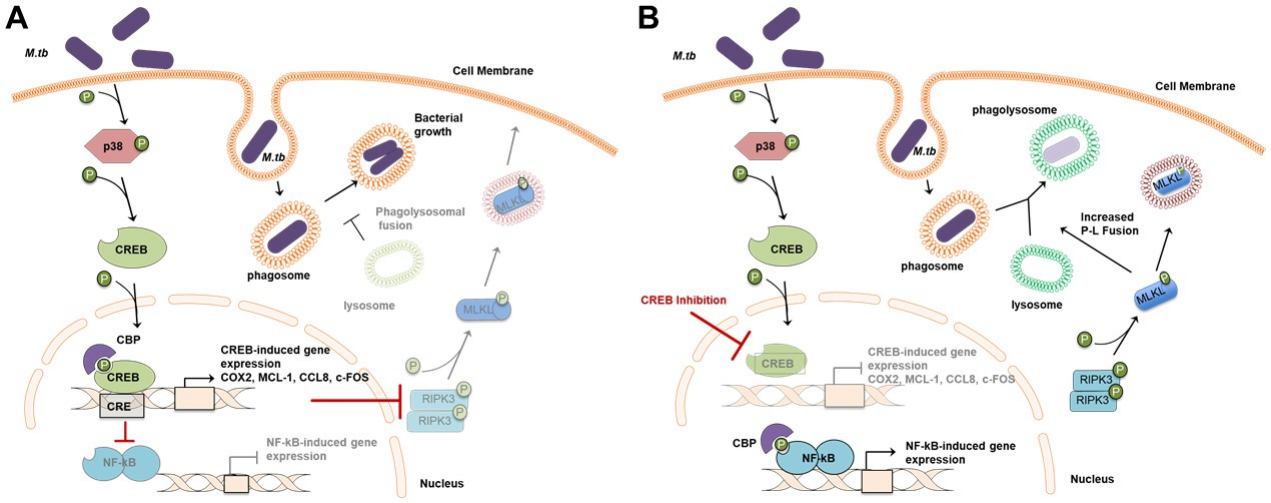
Model. In human macrophages, *M*.*tb* infection induces phosphorylation of p38 MAPK resulting in downstream phosphorylation of the transcription factor CREB. A) Phosphorylated CREB enters the nucleus and forms a transcriptional complex binding coactivator CREB binding protein (CBP), corresponding with decreased nuclear colocalization of NF-kB, which also requires CBP [10]. Activated CREB transcribes *M*.*tb*-induced genes including early expression of COX2, MCL-1, CCL8 and c-FOS. CREB also prevents phosphorylation of RIPK3 and MLKL (red blocking arrow) limiting pMLKL-dependent lysosomal fusion with the *M*.*tb*-containing phagosome, all things that contribute to intramacrophage bacterial growth. B) When CREB binding to CBP is inhibited with 666–15 constraining CREB signaling (red blocking arrow), less CREB is localized to the nucleus. In contrast, NF-kB nuclear localization is increased during CREB inhibition. It is possible that CBP is now available to bind NF-kB, likely leading to increased transcription of NF-kB regulated gene expression. CREB inhibition also leads to increased phosphorylation of RIPK3 and MLKL, resulting in increased phagolysosomal fusion (P-L fusion) corresponding with decreased bacterial growth in human macrophages. Phosphorylated MLKL can be expelled in extracellular vesicles or tagged for proteosomal or lysosomal degradation (pMLKL in red endosome), preventing necroptosis and macrophage death [38,43].

We have identified CREB as a critical effector of immune evasion used by *M*.*tb* to counteract certain antimicrobial mechanisms in human macrophages. In this respect, we show that *M*.*tb* induces phosphorylation and nuclear translocation of CREB via the p38-MAPK signaling pathway in the first minutes to hours post-infection, dampening macrophage NF-kB translocation and increasing transcription of certain CREB-regulated IEGs. We also show for the first time that CREB negatively regulates phagolysosomal fusion during *M*.*tb* infection by restricting activation of RIPK3-induced pMLKL, thus providing another potential mechanism allowing for bacterial growth in human macrophages (Fig 11).

Previous reports have suggested that CREB is important for *M*.*tb* pathogenesis. Work using cells from human patients showed that CREB binds to the IFN-γ proximal promoter positively regulating IFN-γ production in T cells in patients with latent *M*.*tb* infection (LTBI) or healthy tuberculin reactors [44,45]. However, patients with active TB have less phosphorylated CREB and less nuclear CREB compared to LTBI or healthy individuals [44,45]. These studies show a protective role for CREB in T cells to defend against TB. In contrast, CREB activity in macrophages is associated with *M*.*tb* pathogenesis and is beneficial to bacterial survival. A hypervirulent *M*.*tb* mutant lacking sigma factor sigI induced more pCREB compared to the parent strain CDC1551 in J774.1 cells [46]. In addition, CREB knockdown with siRNA in RAW macrophages led to decreased *M*.*tb* growth [14]. These studies point to a detrimental role for CREB in host control of *M*.*tb* in murine macrophages and cell lines, however, the function of CREB in human macrophages in response to virulent *M*.*tb* was previously unknown.

Investigating macrophage cAMP signaling during *M*.*tb* infection is complicated by the ability of the bacteria to produce cAMP and modulate protective host signaling pathways [8,12,47]. In earlier work, Agarwal et al. noted increased cAMP levels and phosphorylated CREB in J774 cells post-infection with *M*.*tb* strain CDC1551 in an MOI-dependent response [12]. The source of cAMP was determined to be the bacteria. In the current study, we used primary human MDMs infected with a lower MOI of *M*.*tb* H_37_R_v_ as well as CDC1551 and other mycobacterial strains and were unable to detect a significant increase in cAMP levels above uninfected cells using our assay. Nevertheless, we observed significantly more pCREB in response to *M*.*tb* infection. It is possible that the difference noted between our study and Agarwal et al. is the assay or macrophage system used, i.e., mouse versus man and/or primary vs cell line. However, our data also show that inhibition of p38 abolishes *M*.*tb*-induced pCREB to levels consistent with uninfected MDMs, further demonstrating that *M*.*tb*-induced pCREB activation in human macrophages is cAMP independent and reliant on the p38 MAPK pathway. We also initiated investigations on the *M*.*tb*-receptor interaction(s) that may be responsible for engaging the p38 signaling pathway to activate CREB. Early data suggest a role for toll-like receptor 2 in CREB phosphorylation in human macrophages in response to *M*.*tb*, however the data were inconsistent. Additional studies are underway to determine the receptor(s) required for *M*.*tb*-induced CREB activation.

The inflammatory response in macrophages and in tissues is tightly regulated by the activities of transcription factors such as CREB and NF-kB. CREB regulates numerous cell signaling pathways in macrophages, most commonly associated with promoting anti-inflammatory or immunoregulatory environments [10]. NF-kB, on the other end of the inflammatory spectrum, is largely responsible for promoting a pro-inflammatory, antimicrobial environment and is associated with protection against *M*.*tb* infection in macrophages and in mouse models [32–34], although conflicting data exist for human macrophage control of *M*.*tb* [48], demonstrating a high level of complexity in the NF-kB signaling pathways. We show here that infection of MDMs with *M*.*tb* induces nuclear translocation of NF-kB p65. It has been proposed that NF-kB and CREB compete for the coactivator CBP/p300 due to the finding that the RelA/p65 component of NF-kB interacts with the same regions of CBP/p300 as CREB [10,35]. Consistent with this, we show that inhibition of CREB activity allowed for increased and prolonged nuclear NF-kB p65 in *M*.*tb*-infected human macrophages. This indicates that CREB is restrictive of NF-kB’s presence in the nucleus, likely as a counter to the proinflammatory program characteristic of NF-kB. Our data suggest that *M*.*tb* induces CREB activation, in part, to negate the anti-mycobacterial activities associated with NF-kB, allowing for intramacrophage bacterial growth.

A critical role of CREB is induction of IEGs to rapidly respond to stimuli, including bacterial infection. We highlight that CREB signaling is important for early expression of COX2, MCL-1, CCL8 and c-FOS. COX2 transcription is regulated by various transcription factors, including CREB, NF-kB and activator protein 1 (AP-1) [49]. *M*.*tb* infection induces COX2 transcription and its enzymatic activity, resulting in production of PGE_2_. In mouse models, PGE_2_ is important for host resistance to infection [27,28], likely due to the immunosuppressive effects of PGE_2_ to limit inflammatory damage rather than an antimicrobial effect. In MDMs, we found that *M*.*tb* infection rapidly induced COX2 gene expression and protein production within the first 3h post-infection in a CREB-dependent manner, resulting in decreased PGE_2_ secretion at 6h. However, it is possible that another transcription factor may compensate for the absence of CREB signaling, boosting COX2 production at a later time point to generate PGE_2_ and prevent an overexuberant inflammatory response. Altogether, these data further demonstrate the important role for the initial macrophage response to *M*.*tb*, which can then influence the outcome of disease [4].

CREB is also known to induce pro-survival factors in macrophages, including MCL-1 [22]. Our lab has previously shown that *M*.*tb* induces MCL-1 in human MDMs to restrict apoptosis in a manner dependent on PPARγ [25]. In the current study, we found that *M*.*tb* infection begins to induce MCL-1 gene expression at 1h post-infection and protein expression as early as 3h post-infection. Our previous observations with siRNA silencing of PPARγ showed a decrease in *M*.*tb*-induced MCL-1 gene expression beginning at 6h post-infection [25]. This suggests that the kinetics for CREB and PPARγ regulation of MCL-1 are different, with CREB-inducing MCL-1 expression early (within the first 1-3h) and PPARγ inducing expression later (6h+).

CCL8 is a proinflammatory chemokine chemotactic for various immune cells. Protein secretion of CCL8 was previously shown to increase upon *M*.*tb* infection in various macrophage models and in pleural effusions of TB patients [50]. CCL8 has also been identified as a potential biomarker for TB [50–53] and is highly expressed in human alveolar macrophages isolated from TB patients [53]. Induction of c-FOS gene transcription was also previously shown to be induced by *M*.*tb* infection [54] and is regulated by CREB in response to external stimuli [23]. Along with c-JUN and others, c-FOS is a component of the AP-1 transcription factor complex, responsible for regulating a diverse set of immunomodulatory genes that have largely been studied in the context of cancer [55]. In RAW macrophages infected with *Brucella abortus*, c-FOS regulated both pro- and anti-inflammatory cytokines, including IL-1β, IL-6 and IL-10 [56]. AP-1/c-FOS was also linked to regulation of IL-27 subunit p28 during *M*.*tb* infection of mouse macrophage-like cell lines [57], suggesting a host-protective role for c-FOS. Both CCL8 and c-FOS are known to modulate various immune mechanisms and further investigation into their role in *M*.*tb*-infected human macrophages is needed.

Programmed cell death (PCD) mechanisms can be used by the host as a defense mechanism but have also been co-opted by pathogens to favor their own survival [58–60]. In the *M*.*tb* field, the data strongly suggest that *M*.*tb* prevents apoptosis, in part through induction of MCL-1, preserving its niche and evading the host’s immune system [25,29,61–63]. Recent reports have further investigated apoptosis and other modes of PCD, including ferroptosis, pyroptosis and necroptosis [58,59,64]. Agonist-induced necroptosis is typically evident by 6-8h post stimulation [42,65]. Concurrent with *M*.*tb* infection, we observed that CREB inhibition resulted cell swelling, suggestive of necroptosis, accompanied by a significant increase in phosphorylation of RIPK3/MLKL. However, treatment with 666–15 did not result in a significant loss in cell viability through 72h post-treatment, indicating that we were not observing PCD during the course of our study. Our data, instead, point to an as yet undescribed role for CREB in repression of phosphorylation of these proteins, and inhibition of a non-necroptotic function for the classical necroptotic signaling pathway.

Work by others investigating RIPK3 and MLKL as potential targets for HDT against *M*.*tb* have had mixed results. Generally, inhibition of or deficiency in RIPK3 or MLKL in vitro resulted in decreased CFUs and increased macrophage viability compared to controls [66,67]. Using a RIPK3 deficient mouse model, Zhao et al. demonstrated that RIPK3 is important for *M*.*tb* growth in vivo, in part due to increased macrophage susceptibility to necrosis [66]. In contrast, Stutz et al, did not detect a change in CFUs between *M*.*tb* infected WT and RIPK3^-/-^ or MLKL^-/-^ mice [68,69]. The authors show that although the necroptosis pathway is primed due to increased total MLKL protein in BMDMs or murine alveolar macrophages infected with *M*.*tb*, necroptosis is not occurring [69].

Recent reports have indicated that macrophages utilize MLKL in an antimicrobial manner that does not induce necroptotic activity [38,70]. Phosphorylated MLKL is targeted to endosomes through site-specific ubiquitination and facilitated endosomal trafficking independent of necroptosis to self-restrict cell death by release of pMLKL in extracellular vesicles [38,43]. Ubiquitinated pMLKL also enhanced trafficking of *L*. *monocytogenes* to lysosomes, resulting in decreased bacterial burden in HT-29 cells and L929 cells treated with agonists to induce necroptosis [38]. Here, we find that inhibition of *M*.*tb*-induced CREB activation results in increased phagolysosomal fusion that is dependent on phosphorylation of MLKL by RIPK3. This increased fusion corresponds to increased macrophage control of bacterial growth during CREB inhibition. *M*.*tb* is known to inhibit fusion of the *M*.*tb* containing phagosome with lysosomes, preventing its own destruction [39–41]. Other mechanisms have been shown to impair *M*.*tb* trafficking to the lysosomes, including certain *M*.*tb* cell wall components and secreted proteins that modulate macrophage cell signaling [71,72]. Here, we add to this list, implicating a cell-signaling pathway that has been understudied in the context of *M*.*tb* infection. These data are the first to show that *M*.*tb* activates CREB to impede bacterial trafficking by preventing phosphorylation of MLKL.

Through the use of inhibitors and siRNA, our data suggest that activated RIPK3/MLKL plays a host-protective function within the first 2h of *M*.*tb* infection due to increased phagolysosomal fusion. However, longer term inhibition of RIPK3/MLKL (72h post-infection) has previously been shown to extend cell viability by inhibiting necroptosis, coinciding with increased control of *M*.*tb* growth [66,67]. Owing to these additional effects of GSK’872 and MLKL knockdown at later time points, we were unable to ascertain whether the increased phagosome-lysosome fusion induced by CREB inhibition and pMLKL at 2h post-infection was directly responsible for the decreased bacterial growth that results from CREB deficiency at later time points. It is likely that CREB inhibition results in multiple changes in the cell that work in concert to restrict bacterial growth. Additional studies are ongoing to fully examine CREB’s role in human macrophages infected with *M*.*tb*.

Our findings implicate CREB, a master regulator of macrophage cell signaling, as a critical factor in immune evasion by *M*.*tb* in human macrophages (Fig 11). Previous studies in our lab showed that freshly isolated human alveolar macrophages express gene transcripts for COX2, MCL-1, RIPK3 and MLKL [73]. *M*.*tb* infection was also shown to increase MCL-1 gene expression and protein production in human AMs [25] suggesting that our findings can potentially translate to *M*.*tb* infection in human lungs. Curbing the antimicrobial responses in the first minutes to hours post-infection likely shapes the immune response to *M*.*tb*, allowing the bacteria to claim a foothold in the macrophage and establish its niche. The initial response to bacterial invasion by alveolar macrophages is key to limited bacterial spread in the airways and Th1 priming leading to bacterial control [4,5]. Inhibition of CREB signaling is likely to result in activation of redundant pathways, including certain roles for NF-kB and CREB family members cAMP response element modulation protein (CREM) and activating transcription factor 1 (ATF1). Thus, targeting CREB itself as an HDT for TB is less certain to have a beneficial effect since it would likely induce off-target effects. However, identification and understanding of key signaling pathways regulated by CREB during *M*.*tb* infection could result in attractive, more specific, targets for HDT and add to our arsenal to fight TB.

## Materials and methods

### Ethics statement

Peripheral blood mononuclear cells (PBMCs) were isolated from human peripheral blood collected from healthy donors, following Texas Biomedical Research Institute protocols approved by the UT Health San Antonio Institutional Review Board, protocol number 2017315HU. All donors for these studies provided informed, written consent.

### Isolation and culture of human monocyte-derived macrophages (MDMs)

MDMs were prepared as described elsewhere [74]. Briefly, heparinized blood was layered on a Ficoll-Paque cushion (GE Healthcare, Uppsala, Sweden) to allow for collection of PBMCs. PBMCs were cultured in RPMI (Life Technologies, Carlsbad, CA) with 20% autologous serum in Teflon wells (Savillex, Eden Prairie, MN) for 5 days at 37°C/5% CO_2_. MDMs were harvested and adhered to tissue culture dishes for 2–3 h in RPMI with 10% autologous serum, lymphocytes were washed away, and MDMs were incubated overnight in RPMI with 10% autologous serum. Such MDM monolayers are 99% pure and viable.

### Bacterial strains

Lyophilized *M*.*tb* H_37_R_v_ (ATCC# 27294), *M*.*tb* H_37_R_a_ (ATCC# 25177), *M*. *bovis* BCG (ATCC# 35734), and *M*. *smegmatis* (ATCC# 700084) were obtained from the American Type Culture Collection (ATCC, Manassas, VA). GFP-*M*.*tb* Erdman was a kind gift from Dr. Marcus Horwitz, UCLA, CA). Clinical isolates CDC1551 and HN878 were from BEI Resources (Manassas, VA). *M*.*tb* H_37_R_v_-mCherry was kindly provided by Dr. Sarah Fortune (Harvard University). Single cell suspensions of bacteria were prepared as previously described [75,76]. The bacteria concentration and degree of clumping (<10%) were determined with a Petroff-Hausser Chamber. This method results in approximately 90% viable bacteria, as determined by CFU assay.

### *M*.*tb* infection of macrophages

Single cell suspensions of *M*.*tb* in RHH [10mM HEPES (Life Technologies) and 0.1% human serum albumin (CSL Behring, King of Prussia, PA) in RPMI] were added to macrophages at various MOIs, centrifuged at 350xg for 5 min at 4°C, and then incubated at 37°C with 5% CO_2_ to synchronize phagocytosis. For CFU assays and PGE_2_ assay, infected cells were incubated for 2h at 37°C, with the first 30 min on a platform shaker. Macrophages were then washed and incubated in RPMI with 2% autologous serum for the indicated times. Where indicated, MDMs were pre-treated for 1h with solvent controls (DMSO), or reagents Forskolin, PGE_2_, IBMX, SB203580, UO126, 666–15 +/- necrostatin-1, GSK’872 or necrosulfonamide prior to infection (S1 Table). All inhibitors were maintained throughout the course of infection.

### siRNA Transfection

Briefly, MDMs were seeded to tissue cultures plates as described above in RPMI supplemented with 10% autologous serum. MDMs were transfected with scrambled siRNA (100 nM; D-001810-10) or MLKL siRNA (100 nM; L-005326-00-0005) from Dharmacon (Lafayette, CO) or scrambled siRNA (150 nM; sc-37007) or CREB1 siRNA (150 nM; sc-29281) from Santa Cruz Biotechnology (Dallas, TX) using the TransIT-X2 Dynamic Delivery System (Mirus, Madison, WI) according to manufacturer’s instructions. Transfected MDMs were then incubated for 48h (siMLKL) or 72h (siCREB) at 37°C/5% CO_2_. Knockdown of MLKL and CREB was verified by Western blot.

### Western blotting

Cells were washed with PBS, then lysed with TN1 lysis buffer (125 mM NaCl, 50 mM Tris, 10 mM EDTA, 1% Triton X-100, 10 mM Na_4_PO_7_, 10 mM NaF with 10 mM Na_3_VO_4_, 10 μg/ml aprotinin, and 10 μg/ml leupeptin) at 4°C. Lysates were centrifuged (10,000g, 4°C, 10 min) to remove cell debris, then a Pierce BCA assay (Thermo Scientific, Waltham, MA) was performed to determine protein concentration. Equivalent amounts of denatured and reduced protein were separated by SDS-PAGE and analyzed by Western blot using antibodies in S1 Table. Immunoblots were imaged using VisionWorks software on the UVP ChemStudio (Analytik Jena, Upland, CA). Protein band intensities were determined with VisionWorks software, for each sample background values were subtracted and then values were normalized to the β-actin loading control. Phosphorylated proteins were normalized to total protein.

### Confocal microscopy

Day 5 MDMs were plated onto Chromerge-cleaned glass coverslips in 24-well tissue culture plates for 2h at 37°C, washed to remove non-adherent cells and repleted with RPMI supplemented with 10% autologous serum, as described above. At specified time points post-infection, MDM monolayers on coverslips were washed with PBS, fixed with 4% paraformaldehyde, and permeabilized with 100% ice-cold methanol for 2 min at room temperature. The cells were blocked overnight at 4°C in blocking buffer (5 mg/ml BSA, 10% heat-inactivated FBS in Dulbecco’s PBS), incubated with primary Abs (S1 Table), followed by incubation with fluorophore conjugated secondary Abs. For isotype control, the permeabilized MDMs were incubated with mouse or rabbit IgG as appropriate. MDM nuclei were labeled with 0.1mg/ml of the DNA stain DAPI (Molecular Probes, Carlsbad, CA) in PBS for 10 min at room temperature. After extensive washing, the coverslips were mounted on glass slides. Immunofluorescence was examined by confocal microscopy (ZEISS LSM 800 Confocal Laser Scanning Microscope, White Plains, NY). Enumeration and fluorescence were quantified with ImageJ Software where indicated. Bacterial association with MDMs was assessed manually by counting mCherry-expressing *M*.*tb* H_37_R_v_ associated with macrophages at 20x magnification and normalized to the total number of macrophages. *M*.*tb* colocalization with LAMP-1 was assessed by manual counting at 63x magnification and percent of bacteria colocalizing with LAMP-1 was calculated. At least 100 bacteria in over 100 macrophages were counted per condition.

### RNA isolation and gene expression by qRT-PCR

Macrophages in duplicate or triplicate wells were lysed with TRIzol (Invitrogen) and total RNA was isolated according to the manufacturer’s recommendations. The NanoDrop One (Thermo Scientific) was used to determine quantity and quality of RNA. cDNA was reverse transcribed from RNA with SuperScript III Reverse Transcriptase (Invitrogen). Gene expression was determined by quantitative real-time PCR (qRT-PCR) using TaqMan Gene Expression Assays (Applied Biosystems, Foster City, CA) and an Applied Biosystems 7500 Real-Time Machine, QuantStudio 5 Real-Time PCR System (Thermo Scientific) or QuantStudio 6 Real-Time PCR System. Values were normalized to β-actin, which was used as a housekeeping gene using the ΔΔCt method [77,78].

### PGE_2_ ELISA

MDMs were incubated with the CREB inhibitor for 1h prior to addition of *M*.*tb* at MOI 5. After 6h, cell free supernatants were collected and the amount of PGE_2_ in the supernatant was analyzed with a PGE_2_ EIA kit (Cayman Chemical, Ann Arbor, MI) according to the manufacturer’s instructions.

### cAMP assay

MDMs were incubated +/- DMSO or IBMX (100 μM) for 1h, then incubated with *M*.*tb* at MOI 5 or 10, PGE_2_ (0.1 μM) or forskolin (50 μM). After 15 min, 30 min, 1h or 24h, supernatants were aspirated and cells were frozen for 2-4h at -80°C to lyse cells. Lysates were collected by adding cAMP ELISA buffer and scraping, then clarified by centrifugation (10,000xg, 4°C, 10 min) and the amount of cAMP in the lysates was analyzed with a cAMP EIA kit (Cayman Chemical) according to the manufacturer’s instructions.

### *M*.*tb* growth assays

Supernatant was removed from infected MDMs and centrifuged at 100xg for 10 min to pellet non-adherent, viable macrophages. Extracellular bacteria in the supernatant were discarded, then pelleted cells were resuspended in 7H9 broth (BD Biosciences, Franklin Lakes, NJ). Concurrently, cold DNase (Millipore-Sigma, Burlington, MA) was added to the adherent monolayer. After 10 min with intermittent shaking, pelleted non-adherent macrophages in 7H9 broth were added back to the wells containing DNAse and the adherent macrophages. Macrophages were then lysed with 0.25% sodium dodecyl sulfate (SDS, FisherScientific) in phosphate-buffered saline for 10 min, then 20% BSA (ThermoFisher) in sterile water was added. Lysates were diluted and plated on 7H11 agar (Remel, San Diego, CA). The number of CFUs was enumerated after growth for 3–4 weeks at 37°C.

### MDM monolayer integrity and cell viability

To assess monolayer integrity during the course of experiments, at least three images per condition were acquired under 40x magnification with phase microscopy (Olympus DP71 microscope digital camera or EVOS XL Core Imaging System, ThermoFisher Scientific). Membrane integrity and cell viability was assessed by CytoTox-Glo Cytotoxicity Assay and LDH-Glo Cytotoxicity Assay according to manufacturer recommendations in a 96 well plate using the GloMax Navigator microplate reader (Promega).

### Statistical analysis

Macrophages from at least three different donors were used for each assay, unless indicated otherwise. Although the trend was the same for each donor, the magnitude of change differed among donors. Consequently, results from each experiment were normalized to an internal control where indicated and an unpaired one-tailed Student’s t-test or ANOVA were performed on the normalized data using Graphpad (San Diego, CA), with p < 0.05 considered significant.

## Supporting information

S1 Fig Treatment with 666–15 inhibits CREB nuclear colocalization. MDMs were plated on glass coverslips and pretreated with DMSO or the CREB inhibitor, 666–15, for 60 min then infected with *M*.*tb* H_37_R_v_ at MOI 10. At 1h post-infection, cells were fixed, permeabilized, and stained for pCREB (red) and DAPI in the nucleus (blue). A) MDMs were imaged at 20x and a representative experiment is shown of n = 5 donors. B) MFI of pCREB signal that colocalized with DAPI (magenta) was calculated using ImageJ Fiji software, normalized to total number of cells per field and graphed as fold change ± SEM compared to infected, cells. Data are cumulative of n = 5 donors. C) MDMs were pretreated as described and infected with mCherry *M*.*tb* H_37_R_v_. Bacteria associated with MDMs were counted and normalized to total cell number in each field and graphed as fold change ± SEM compared to *M*.*tb* infected cells. Data are cumulative of n = 4 donors; *p < 0.05, **p < 0.01.
(TIF)

S2 Fig Effect of *M*.*tb* infection and CREB inhibition on expression of select genes and c-FOS protein. MDMs were pretreated with DMSO or 666–15 for 60 min and subsequently infected with *M*.*tb* H_37_R_v_ at the MOI 10 by synchronized phagocytosis. A) RNA was collected at the indicated time points and gene expression of the indicated genes was determined by qRT-PCR. Data are shown as fold change ± SEM compared to uninfected MDMs. Data are cumulative of n = 3–5 donors. One-way ANOVA with Tukey’s post-test. B) Cell lysates were probed by WB for c-FOS and β-actin. Shown is a representative experiment of n = 3. C) Densitometry at 3h post infection compared to *M*.*tb*-infected MDMs. Data are cumulative ± SEM of n = 3 donors. Unpaired t test; *p < 0.05, **p < 0.01, ***p < 0.001.
(TIF)

S3 Fig Phosphorylation of CREB in human macrophages based on *M*.*tb* MOI. MDMs were infected with *M*.*tb* H_37_R_v_ by synchronized phagocytosis at the indicated MOI. A) Western blot was performed to detect levels of phosphorylated and total CREB protein at 1h post-infection. WB is representative of n = 2 donors. B) Densitometry analysis was performed and ratios of pCREB/total CREB were determined. Data are cumulative ± SEM of n = 2 donors. One-way ANOVA with Tukey’s post-test; *p < 0.05, **p < 0.01.
(TIF)

S4 Fig CREB inhibition does not result in significantly increased LDH release. MDMs were pretreated with 666–15 or DMSO control for 60 min and subsequently infected with *M*.*tb* H_37_R_v_ at MOI 2. Membrane integrity and cell viability was determined by LDH assay. Data are cumulative ± SEM of n = 3–4 donors. Two-way ANOVA with Tukey’s post-test.
(TIF)

S5 Fig Inhibition of the necroptotic signaling pathway is not sufficient to affect phagolysosomal fusion in *M*.*tb*-infected human macrophages. A) MDMs were plated on glass coverslips and pretreated for 60 min with DMSO or CREB inhibitor 666–15 +/- Nec-1, GSK’872, or NSA, then infected with mCherry *M*.*tb* H_37_R_v_ (red) MOI 10. MDMs were fixed, permeabilized, and stained for LAMP-1 (green) and DAPI (blue). A representative experiment is shown of n = 2–3 donors. B) At 2h post-infection, the percent of *M*.*tb* colocalizing with LAMP-1 was calculated following manual counting. White arrows indicate colocalization. Data are representative ± SD of n = 2–3 donors. One-way ANOVA with Tukey’s post-test; *p < 0.05, **p < 0.01, ***p < 0.001, ****p < 0.0001.
(TIF)

S1 Table Reagents and Antibodies. (PDF)
