# Supplementary figures and images for: Activation of transcription factor CREB in human macrophages by Mycobacterium tuberculosis promotes bacterial survival, reduces NF-kB nuclear transit and limits phagolysosome fusion by reduced necroptotic signaling
Source: PLoS Pathog. 2023 Mar 31;19(3):e1011297. doi: 10.1371/journal.ppat.1011297 (PMC10096260; doi:10.1371/journal.ppat.1011297)

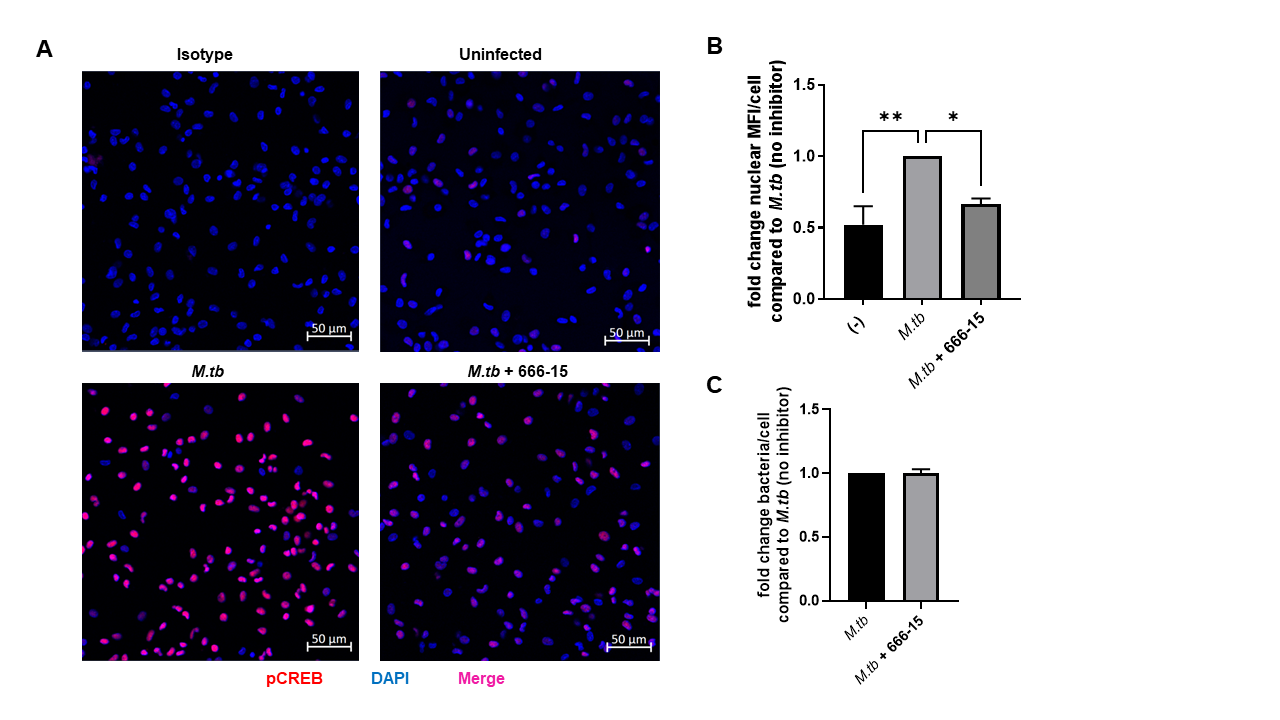

Supplement: S1 Fig — MDMs were plated on glass coverslips and pretreated with DMSO or the CREB inhibitor, 666–15, for 60 min then infected with M.tb H37Rv at MOI 10. At 1h post-infection, cells were fixed, permeabilized, and stained for pCREB (red) and DAPI in the nucleus (blue). A) MDMs were imaged at 20x and a representative experiment is shown of n = 5 donors. B) MFI of pCREB signal that colocalized with DAPI (magenta) was calculated using ImageJ Fiji software, normalized to total number of cells per field and graphed as fold change ± SEM compared to infected, cells. Data are cumulative of n = 5 donors. C) MDMs were pretreated as described and infected with mCherry M.tb H37Rv. Bacteria associated with MDMs were counted and normalized to total cell number in each field and graphed as fold change ± SEM compared to M.tb infected cells. Data are cumulative of n = 4 donors; *p < 0.05, **p < 0.01. (TIF) [file ppat.1011297.s001.TIF]

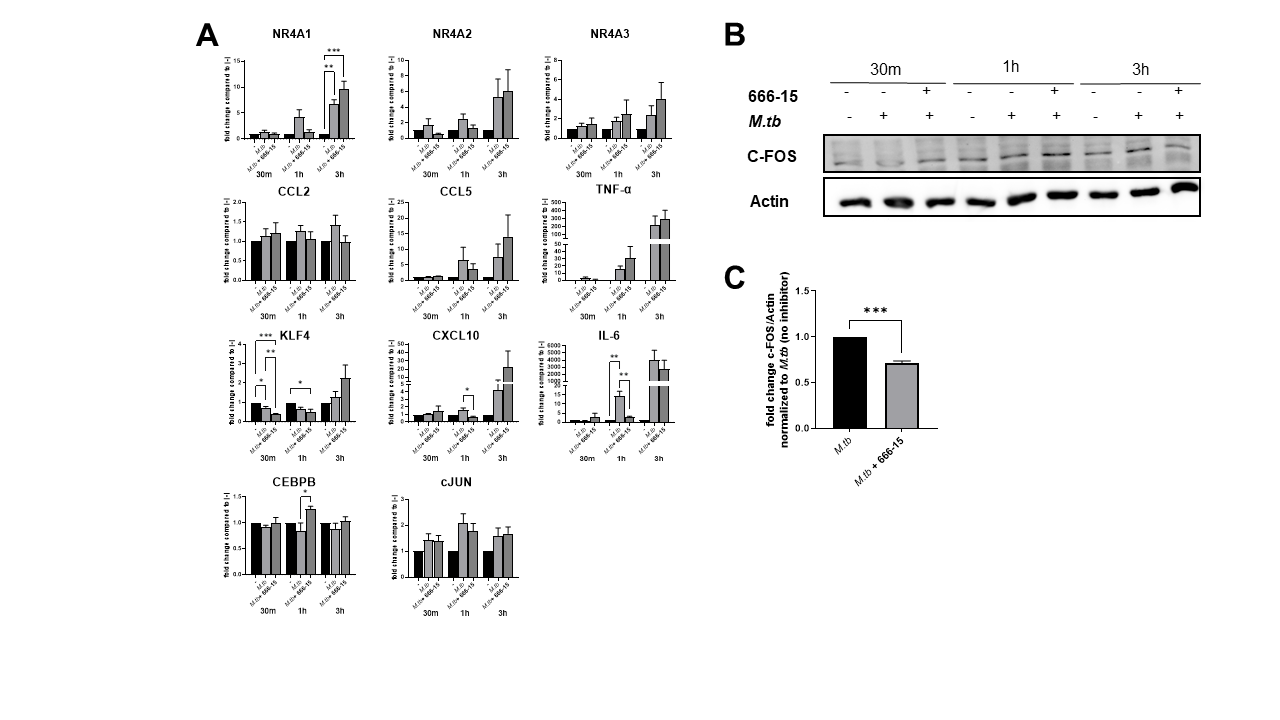

Supplement: S2 Fig — MDMs were pretreated with DMSO or 666–15 for 60 min and subsequently infected with M.tb H37Rv at the MOI 10 by synchronized phagocytosis. A) RNA was collected at the indicated time points and gene expression of the indicated genes was determined by qRT-PCR. Data are shown as fold change ± SEM compared to uninfected MDMs. Data are cumulative of n = 3–5 donors. One-way ANOVA with Tukey’s post-test. B) Cell lysates were probed by WB for c-FOS and β-actin. Shown is a representative experiment of n = 3. C) Densitometry at 3h post infection compared to M.tb-infected MDMs. Data are cumulative ± SEM of n = 3 donors. Unpaired t test; *p < 0.05, **p < 0.01, ***p < 0.001. (TIF) [file ppat.1011297.s002.TIF]

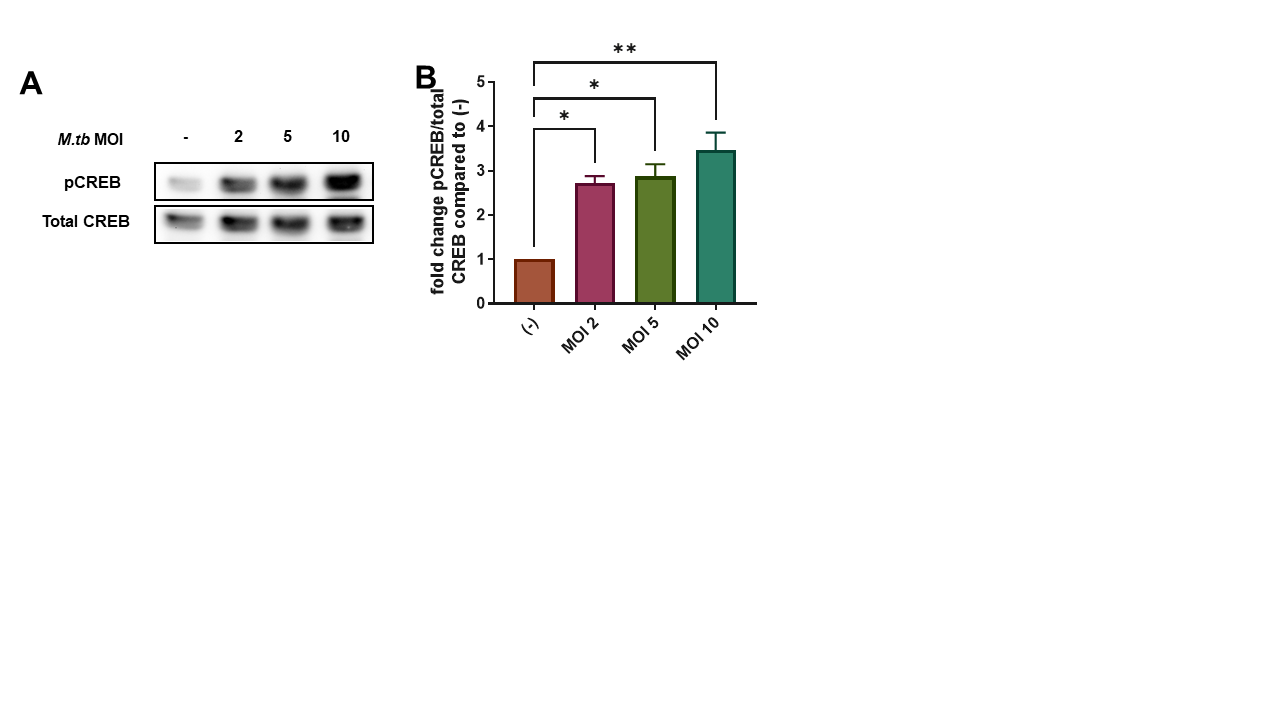

Supplement: S3 Fig — MDMs were infected with M.tb H37Rv by synchronized phagocytosis at the indicated MOI. A) Western blot was performed to detect levels of phosphorylated and total CREB protein at 1h post-infection. WB is representative of n = 2 donors. B) Densitometry analysis was performed and ratios of pCREB/total CREB were determined. Data are cumulative ± SEM of n = 2 donors. One-way ANOVA with Tukey’s post-test; *p < 0.05, **p < 0.01. (TIF) [file ppat.1011297.s003.TIF]

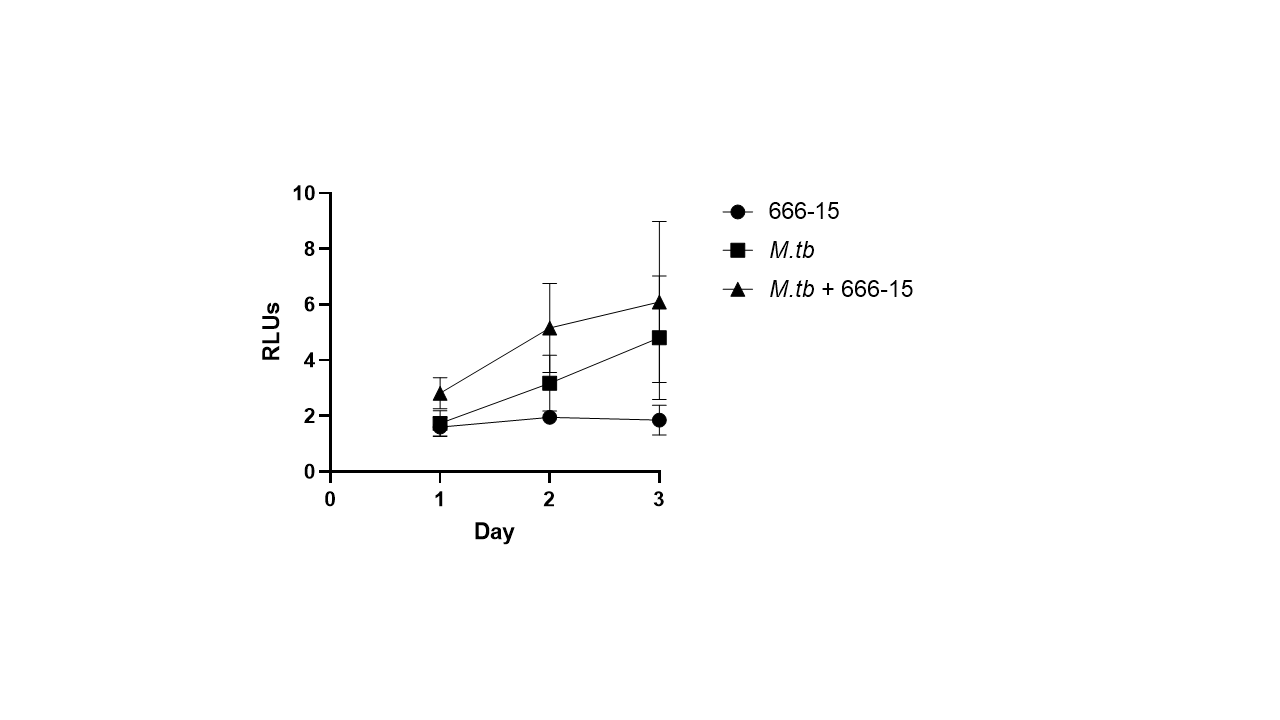

Supplement: S4 Fig — MDMs were pretreated with 666–15 or DMSO control for 60 min and subsequently infected with M.tb H37Rv at MOI 2. Membrane integrity and cell viability was determined by LDH assay. Data are cumulative ± SEM of n = 3–4 donors. Two-way ANOVA with Tukey’s post-test. (TIF) [file ppat.1011297.s004.TIF]

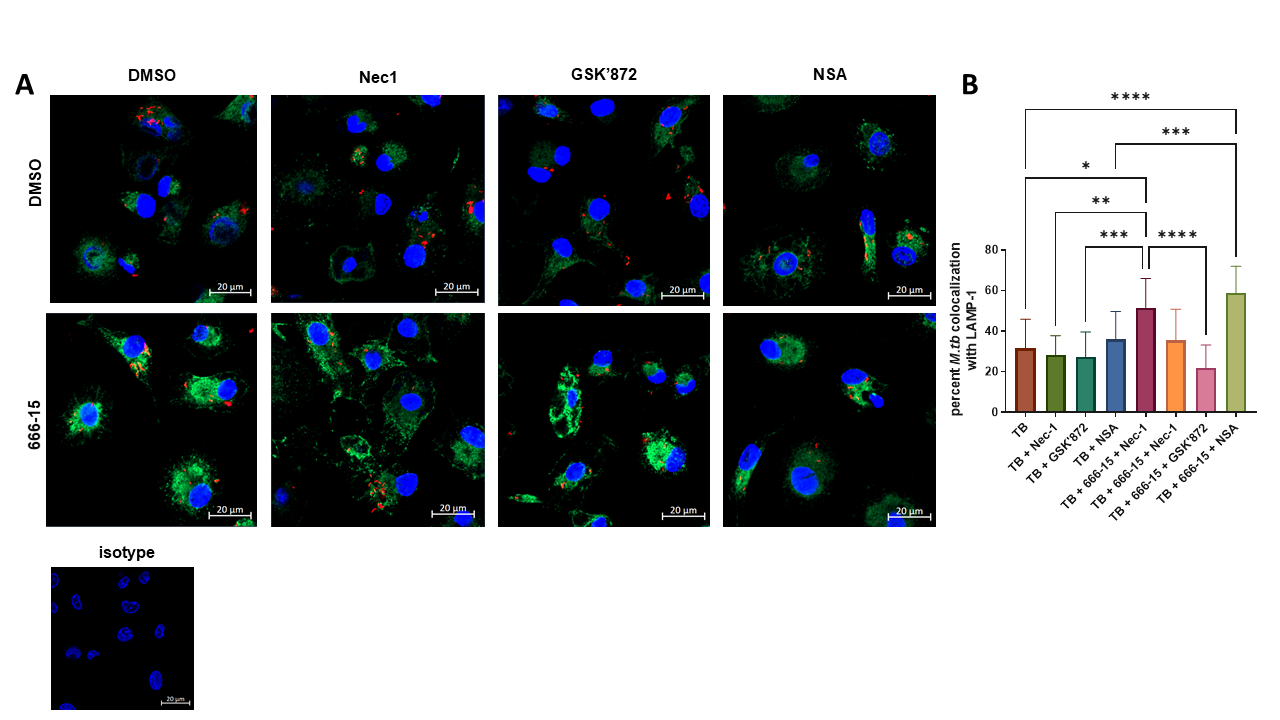

Supplement: S5 Fig — A) MDMs were plated on glass coverslips and pretreated for 60 min with DMSO or CREB inhibitor 666–15 +/- Nec-1, GSK’872, or NSA, then infected with mCherry M.tb H37Rv (red) MOI 10. MDMs were fixed, permeabilized, and stained for LAMP-1 (green) and DAPI (blue). A representative experiment is shown of n = 2–3 donors. B) At 2h post-infection, the percent of M.tb colocalizing with LAMP-1 was calculated following manual counting. White arrows indicate colocalization. Data are representative ± SD of n = 2–3 donors. One-way ANOVA with Tukey’s post-test; *p < 0.05, **p < 0.01, ***p < 0.001, ****p < 0.0001. (TIF) [file ppat.1011297.s005.TIF]
