# Supplementary material for: Activation of transcription factor CREB in human macrophages by Mycobacterium tuberculosis promotes bacterial survival, reduces NF-kB nuclear transit and limits phagolysosome fusion by reduced necroptotic signaling
Source: PLoS Pathog. 2023 Mar 31;19(3):e1011297. doi: 10.1371/journal.ppat.1011297 (PMC10096260; doi:10.1371/journal.ppat.1011297)
Supplement: S1 Table — (PDF) [file ppat.1011297.s006.pdf]

S1 Table: Reagents and Antibodies

**Antibody**	**Vendor**	**Catalog Number**	**Dilution Used**
CREB-HRP	Cell Signaling	99578	1:2000
pCREB	Cell Signaling	9198	1:2000 WB; 1:1000 IF
ERK1/2	Cell Signaling	4695	1:1000
pERK1/2	Cell Signaling	9101	1:1000
p38	Cell Signaling	9212	1:1000
p-p38	Cell Signaling	9215	1:1000
MK2	Cell Signaling	3042	1:1000
pMK2	Cell Signaling	3007	1:1000
COX2	Cayman Chemical	160112	1:1000
B-actin-HRP	Cell Signaling	5125	1:10 000
MCL-1	Santa Cruz	Sc-819	1:500
c-FOS	Cell Signaling	2250	1:1000
p65 NF-kB	Cell Signaling	4764	1:100
LAMP-1	Developmental Studies Hydriboma Bank	H4A3	.25ug/ml
pMLKL	Cell Signaling	91689	1:1000
MLKL	Cell Signaling	14993	1:1000
RIPK1	Cell Signaling	3493	1:1000
pRIPK1	Cell Signaling	65746	1:1000
RIPK3	Cell Signaling	13526	1:1000
pRIPK3	Cell Signaling	93654	1:1000
Isotype Mouse IgG_1	R&D Systems	MAB002	As required
Isotype Rabbit IgG	Cell Signaling	3900	As required
Anti-mouse IgG, HRP-linked Antibody	Cell Signaling	7076	1:5000
Anti-rabbit IgG, HRP-linked Antibody	Cell Signaling	7074	1:5000
Goat anti-rabbit 488	Invitrogen	A11034	1:500
Goat anti-mouse 488	Invitrogen	A11029	1:500
**Agonist/Inhibitor**	**Vendor**	**Catalog Number**	**Concentration Used**
SB203580	Calbiochem	559395	5uM
UO126	Calbiochem	662009	2.5uM
666-15	SelleckChem	S8846	1uM
IBMX	Cell Signaling	13630	100uM
Forskolin	Tocris Bioscience	1099	50uM
PGE_2	Cayman Chemical	14010	0.1uM
Necrosulfonamide	Tocris Bioscience	5025	10uM
GSK’872	MilliporeSigma	5.30389.0001	10uM
Necrostatin-1	MilliporeSigma	5.05224.0001	10uM
DMSO	MilliporeSigma	D2650	As required
